# Inactivating IL34 promotes regenerating muscle stem cell expansion and attenuates Duchenne muscular dystrophy in mouse models

**DOI:** 10.7150/thno.83817

**Published:** 2023-04-23

**Authors:** Yang Su, Yuxin Cao, Chang Liu, Qing Xu, Na Li, Miaomiao Lan, Lei Li, Kun Wang, Zeyu Zhang, Qingyong Meng

**Affiliations:** 1 State Key Laboratories for Agrobiotechnology, College of Biological Sciences, China Agricultural University, Yuanmingyuan West Road No. 2, Haidian District, Beijing 100193, China.; 2 Department of Cell Biology, Third Military Medical University (Army Medical University), Gaotanyan Road No. 30, Shapingba District, Chongqing 400038, China.; 3 State Key Lab of Animal Nutrition, College of Animal Science and Technology, China Agricultural University, Yuanmingyuan West Road No. 2, Haidian District, Beijing 100193, China.; 4 Beijing Advanced Innovation Center for Food Nutrition and Human Health, College of Biological Sciences, China Agricultural University, Yuanmingyuan West Road No. 2, Haidian District, Beijing 100193, China.

**Keywords:** IL34, satellite cells, muscle regeneration, NFKB1, DMD

## Abstract

**Background:** The balance between the differentiation and self-renewal of satellite cells (SCs) is essential for skeletal muscle homeostasis and regeneration. Our knowledge of this regulatory process is incomplete.

**Methods:** Using global and conditional knockout mice as in vivo models and isolated satellite cells as in vitro system, we investigated the regulatory mechanisms of IL34 in the process of skeletal muscle regeneration in vivo and in vitro.

**Results:** Myocytes and regenerating fibers are major source of IL34. Deletion of interleukin 34 (IL34) sustains expansion by sacrificing the differentiation of SCs and leads to significant muscle regeneration defects. We further found that inactivating IL34 in SCs leads to hyperactivation of NFKB1 signaling; NFKB1 translocates to the nucleus and binds to the promoter region of Igfbp5 to synergistically disturb protein kinase B (Akt) activity. Notably, augmented Igfbp5 function in SCs led to deficient differentiation and Akt activity. Furthermore, disrupting Akt activity both in vivo and in vitro mimicked the phenotype of IL34 knockout. Finally, deleting IL34 or interfering Akt in mdx mice ameliorates dystrophic muscles.

**Conclusion:** We comprehensively characterized regenerating myofibers-expressed IL34 plays a pivotal role in controlling myonuclear domain. The results also indicate that impairing IL34 function by promoting SC maintenance can lead to improved muscular performance in mdx mice in which the stem cell pool is compromised.

## Introduction

In adult skeletal muscle, muscle stem cells reside in a special anatomical microenvironment between the basal lamina and sarcolemma in a quiescent state and are therefore named satellite cells (SCs) 1, 2. In response to injury, SCs are initially exposed to a series of damage-associated cues that can trigger cell activation, reentry into the cell cycle to undergo numerous expansions, and eventually differentiation to support the formation of new fibers 3. To sustain long-term skeletal muscle homeostasis, a small population of SCs revert to a quiescent state via a natural biological process named self-renewal to maintain the satellite stem cell pool 4, 5. Cell fate determination, especially decisions on SC differentiation and self-renewal, must be strictly manipulated via a connection between intrinsic and extrinsic cellular regulatory cues during muscle regeneration 6. Abnormal differentiation, self-renewal failure or both can result in poor performance in muscle homeostasis and regeneration 7, 8. During the long-term repair of damaged skeletal muscle, cytokines released by parenchymal and nonparenchymal cells hierarchically regulate SC-associated cellular and molecular events involved in skeletal muscle regeneration 9-11.

Interleukin 34 is an essential cytokine involved in the development and maturation of brain microglia and skin Langerhans cells, and there are few descriptions of its effects on the skeletal muscle system 12. We reported that IL34 is a target of miR-31 in myoblasts, and its forced expression in miR-31-KO SCs results in cell fate determination changes 13. Although IL34 seems to be essential for myogenesis, the role of IL34 in satellite stem cell fate determination and function has not been explored in detail. Nevertheless, our limited knowledge of the contributions of IL34 to muscle regeneration is ascribed to a lack of gain- and loss-of-function investigations in the context of muscle repair.

PI3K-AKT signaling is involved in many essential physiological and pathological processes in animals 14, 15. In response to various growth factors, RKT, a cell membrane receptor, becomes activated and in turn phosphorylated 16. However, there has only been limited research on PI3K-AKT signaling in skeletal muscle regeneration. Exercise protects proliferative SCs against exhaustion via the Igfbp7-Akt-mTOR axis, suggesting that hyperactivation of Akt phosphorylation is bad for satellite stem cell pool maintenance during exercise 17. Downregulated PI3K-AKT signaling through inhibition of PIP5K1α levels in isolated primary myoblasts gives rise to deficient SC differentiation, suggesting that PI3K-AKT is pivotal for myogenic lineage progression of SCs 18. Whether PI3K-AKT signaling plays an important role in the recovery of damaged skeletal muscle and the positive/negative regulatory factors involved in this process are still unknown.

DMD mutation affects 1 in 3,500-5,000 male newborns, mainly resulting in myofibers that are extremely susceptible to injury, leading to consecutive degeneration and regeneration, with eventual SC exhaustion and loss of muscle mass and function 19-23. Furthermore, dystrophin deficiency in SCs causes failure of cell polarity determination and asymmetric division defects, ultimately leading to insufficient generation of myogenic progenitors and impaired regeneration 24. Other studies have suggested that genetic or pharmacological modifications promote SC expansion in mdx mice, leading to improved muscle mass and function 25-27.

Briefly, we show that IL34 is critical for maintaining the balance between SC differentiation and SC self-renewal and sustaining long-term muscle regeneration. IL34 depletion causes increased nuclear translocation of NFKB1 (nuclear factor of kappa light polypeptide gene enhancer in B-cells 1), which binds to the Igfbp5 promoter to augment the expression of Igfbp5, an effective repressor of AKT signaling. Disturbing Igfbp5 in IL34-KO cells can promote SC differentiation, and disturbing AKT activity in WT cells can also impair SC function and muscle repair. We further demonstrate that inactivation of IL34 or pharmacological inhibition of AKT in mdx mice can promote SC expansion and improve muscle function. We propose that targeting IL34 or AKT activity could be a therapeutic approach for ameliorating muscle-wasting diseases.

## Results

### Disrupting IL34 in satellite cells hampers skeletal muscle regeneration

**Figure 1:**
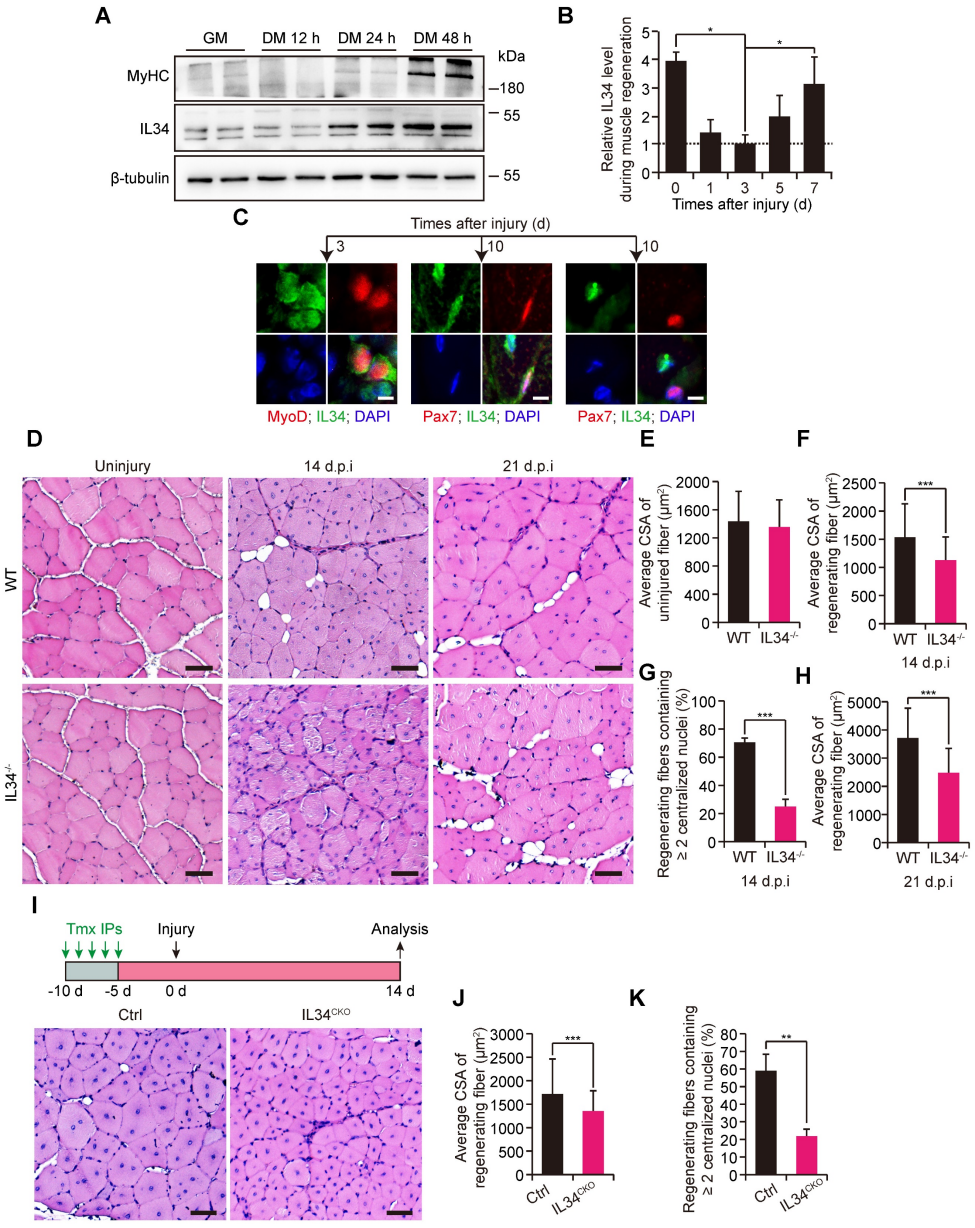
** Inactivation of IL34 hindered long-term skeletal muscle regeneration. (A)** WT SCs were cultured in growth medium for 4 days and then switched to differentiation medium and cultured for 12 h, 24 h or 48 h. Immunoblot analysis of IL34 and MyHC expression in cultured WT SCs at different time points after induction of differentiation. **(B)** Quantitative analysis of IL34 protein levels at various time points in regenerating skeletal muscle (N=3 per group for each time point). *p<0.05. **(C)** Representative images of 3-day injured and 10-day injured TA muscle of WT mice costained for Pax7 and IL34, or MyoD and IL34. The nuclei were labeled using DAPI. Scale bar: 30 μm. **(D)** H&E staining analysis of transverse sections of uninjured and day 14 and 21 postinjury TA muscle from WT and IL34 KO mice. Scale bar: 50 μm. **(E)** Quantification of the average CSA of uninjured TA muscle from WT and IL34 KO mice. **(F)** Quantification of the average CSA of regenerating fibers (14 d.p.i.) TA muscle from WT and IL34 KO mice. N=4 mice in each group. ***p<0.001. **(G)** Quantification of the ratio of regenerating myofibers containing two or more centralized nuclei at day 14 post injury. ***p<0.001. **(H)** Quantification of the average CSA of regenerating fibers (21 d.p.i.) from WT and IL34 KO mice. N=4 mice in each group. ***p<0.001. **(I)** H&E staining analysis of transverse sections from day 14 postinjury of TA muscle from *Ctrl* and *IL34^CKO^* mice. Scale bar: 50 μm. **(J)** Quantification of the fiber size of damaged TA muscle from *Ctrl* and *IL34^CKO^* mice. N=4 mice in each group. ***p<0.001. **(K)** Quantification of the ratio of regenerating myofibers containing two or more centralized nuclei at day 14 post injury. **p<0.01.

We observed that IL34 levels gradually increased in SCs undergoing the process of myogenic lineage progression via immunoblot analysis (Figure 1A). Additionally, IL34 protein was clearly detected in the uninjured TA muscle of adult mice and that the level was dramatically decreased in the 1- and 3-day injured TA muscle of adult WT mice but dramatically restored in the regenerating TA muscle undergoing a period of 5 or 7 days of recovery (Figures 1B and S1A). IL34 had a very low transcription level in quiescent SCs and then was gradually upregulated in SCs during in vitro myogenesis (Figure S1B-D). MyoD^+^ cell.s in 3-day injured TA muscle of adult mice were positive for IL34, Pax7^+^ cells in 10-day injured TA muscle of adult mice were positive or negative for IL34 and around the myonuclei in 10-day injured TA muscle were all positive for IL34 (Figure 1C). This expression pattern indicated that myocytes and regenerating myofibers are major source of IL34.

We measured the regenerative capability of damaged skeletal muscle in adult mice lacking IL34 (Figure S2A-E). The recovery of injured TA muscle exhibited slight changes between WT and IL34-KO mice at the early skeletal muscle regeneration stage (Figure S3).

However, the regenerative capability was significantly blunted in IL34-KO mice relative to WT mice at days 14 and 21 after injury (Figure 1D-H). To clarify that SC-specific deletion of IL34 leads to regeneration deficiency, we employed *Pax7^CreER^*-driven Cre recombinase-mediated conditional knockout mice, in which exons 3-5 of IL34 were flanked by engineered LoxP sites (Figure S2F). Consistently, the skeletal muscle regenerative deficiency of *IL34^CKO^* mice relative to Ctrl mice was also significant when the injured TA muscle underwent a period of 14 days of recovery (Figure 1I-K). Collectively, the results suggest that SC-expressed IL34 is essential for long-term skeletal muscle regeneration.

### Genetic deletion of IL34 increases the SC pool during myogenesis both in vitro and in vivo

**Figure 2:**
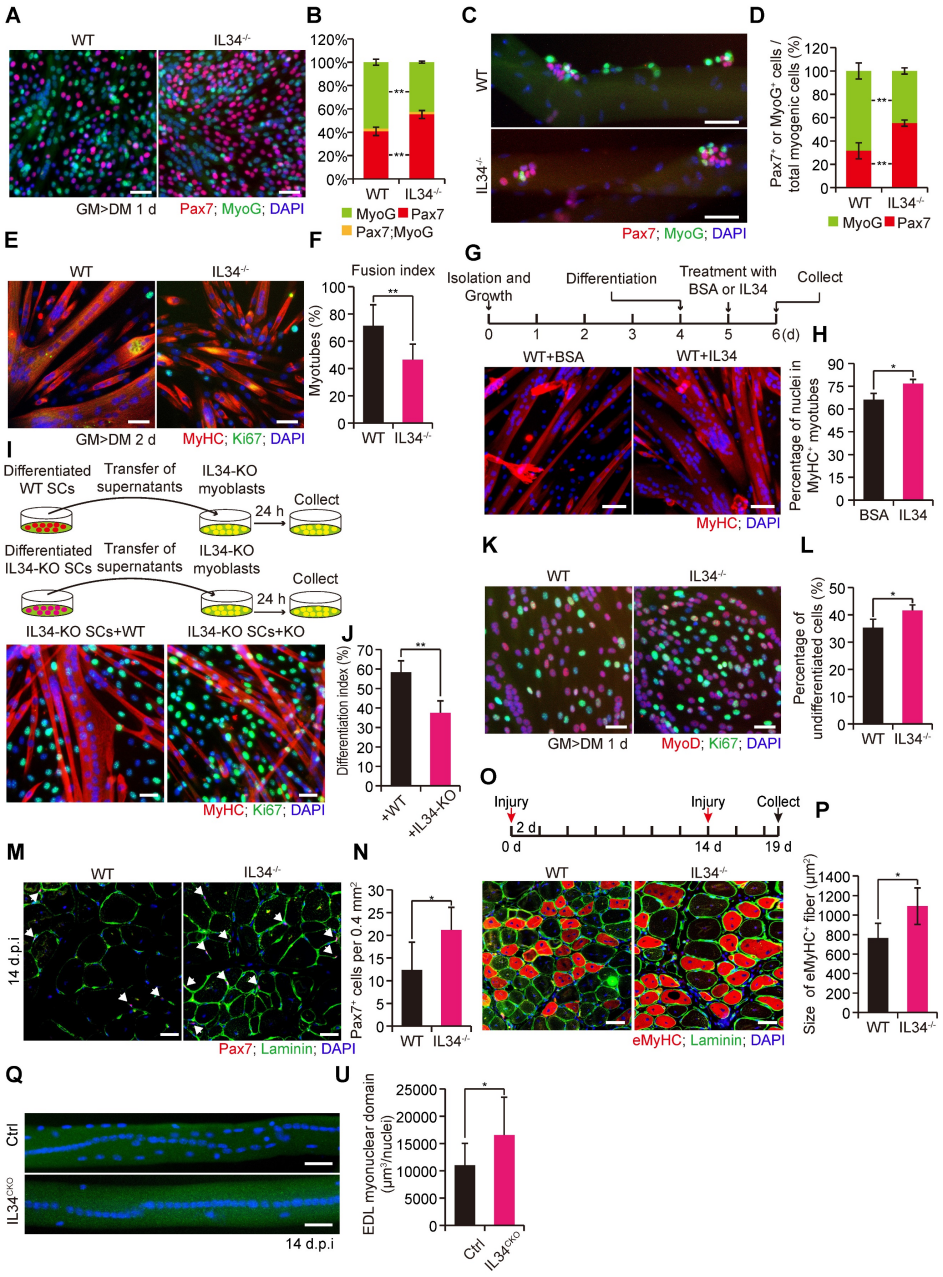
** Inactivating IL34 promotes the expansion and self-renewal of Pax7^+^ cells. (A)** Representative images of WT and IL34-KO SC-induced differentiation after 1 day, with staining for Pax7, MyoG and DAPI. Scale bar: 30 μm. **(B)** Quantification of the percentages of Pax7^+^MyoG^-^, Pax7^+^MyoG^+^ and Pax7^-^MyoG^+^ cell populations in WT and IL34-KO cultures. **p<0.01.** (C)** Representative overlapping images of 72 h cultured myofibers from the EDL muscle of WT and IL34-KO mice costained for Pax7, MyoG and DAPI. Scale bar: 50 μm. **(D)** Percentage of Pax7^+^MyoG^-^ and Pax7^-^MyoG^+^ cell populations in WT and IL34-KO myofiber-associated SCs. **p<0.01. **(E)** WT and IL34-KO SCs were cultured in differentiation medium for 2 days, and representative merged images of cultures were costained with MyHC, Ki67 and DAPI. Scale bar: 30 μm. **(F)** Measurement of the fusion index was calculated by the frequency of MyHC^+^ cells with 2 or more nuclei relative to total MyHC^+^ cells. **p<0.01. **(G)** One day differentiated WT SCs were treated with BSA or IL34 recombinants, and representative merged images of cultures were costained with MyHC and DAPI. Scale bar: 30 μm. **(H)** Measurement of the differentiation index was calculated by the frequency of nuclei in MyHC^+^ cells relative to total nuclei. *p<0.05.** (I)** IL34-KO myoblasts induced to differentiation by supplementing with supernatant medium collected from two day differentiated WT or IL34-KO SCs, respectively. Representative merged images of cultures were costained with MyHC, Ki67 and DAPI. Scale bar: 50 μm.** (J)** Measurement of the differentiation index was calculated by the frequency of nuclei in MyHC^+^ cells relative to total nuclei. **p<0.01. **(K)** FACS-isolated WT and IL34-KO SCs cultured in differentiation medium for 1 day. The cells were then fixed and labeled with MyoD, Ki67 and DAPI. Representative merged photomicrographs of WT and IL34-KO cultures after labeling with MyoD, Ki67 and DAPI. Scale bars: 30 μm. **(L)** Quantitative analysis of the frequency of undifferentiated MyoD^-^Ki67^+^ to MyoD^+^Ki67^+^ cells in WT and IL34-KO cultures. *p<0.05. **(M)** Representative merged images of transverse sections on day 14 postinjury TA muscle from WT and IL34-KO mice stained for Pax7 and laminin. Scale bar: 50 μm. **(N)** Quantification of the number of Pax7^+^ cells per area in WT and IL34-KO mice. *p<0.05. **(O)** After experiencing a period of 14 days of recovery, the damaged muscle was subjected to a second round of injury via injection of 1.2% BaCl_2_. Representative merged photomicrographs of transverse sections of TA muscle 5 days after a second round injury immunostained for eMyHC, laminin and DAPI. Scale bar, 50 μm. **(P)** Quantification of the average CSA of eMyHC^+^ fibers from WT and IL34 KO mice suffering from a second round of damage. N=3 mice in each group. *p<0.05. **(Q)** Single myofibers isolated from 14 day injured EDL muscle of Ctrl and IL34^CKO^ mice and immediately fixed to stain with DAPI. Scale bar, 50 μm.** (U)** Measurement of myonuclear domain between Ctrl and IL34^CKO^ fibers. *p<0.05.

We found that the proliferative capability of SC-inactivating IL34 exhibited no clear changes related to WT SCs (Figure S4). Interestingly, there was a remarkable increase in the percentage of Pax7^+^ cells and a decrease in the percentage of MyoG^+^ cells in IL34-KO differentiated cultures relative to WT differentiated cultures (Figure 2A and B). Consistent with plated SC cultures, myofiber-associated clusters of SCs displayed a markedly higher number of Pax7^+^ cells and a clearly lower number of MyoG^+^ cells on myofibers isolated from IL34-KO mice than on those isolated from WT mice (Figures 2C, D and S5A, B). Consistently, there was also a dramatical increase in the ratio of undifferentiated Ki67^+^ cells in IL34-KO cultures relative to WT cultures grown in neutral medium (10% horse serum) (Figure S6A and B). After 2 days in differentiation medium, we indeed detected differentiation deficiency in IL34-KO SC cultures, as shown by a lower differentiation index relative to that of WT SCs (Figures 2E, F and S6C). This deficiency was also further confirmed by analyzing myogenic lineage progression in cultured WT and IL34-KO SCs (Figure S6D and E). We then treated one day-differentiated WT SCs and IL34-KO SCs with BSA or IL34 recombinants for another 24 hours, and found that IL34-treatment dramatically promoted differentiation of WT SCs and IL34-KO SCs compared to BSA groups (Figures 2G, H and S6F, G). These results indicate that IL34 functions as an essential differentiation agonist.

As newly-formed myofibers are the main contribution of IL34 to undifferentiated Pax7^+^ cells microenvironment, we investigate whether myofiber-transcribed IL34 could affect fate of SCs. We then cultured single myofibers isolated from EDL muscle of *IL34^CKO^* mice, in which IL34 was specifically depleted in Pax7-expressing SCs but not in myofibers. Interestingly, the percentage of Pax7^+^ cell and MyoG^+^ cell residing on myofibers was no evident changes between Ctrl and *IL34^CKO^* mice (Figure S6H and I). We also supplemented IL34-KO SCs and WT SCs with supernatant medium collecting from differentiated WT and IL34-KO cultures for 24 h, respectively. The differentiation index was clearly enhanced in IL34-KO SCs and WT SCs received WT supernatant medium relative to that treated with IL34-KO supernatant medium (Figures 2I, J and S6J, K). This indicates that myotubes-secreted IL34 plays an essential role in inducing the differentiation of SCs.

The proportion of cells that were labeled by EdU was dramatically increased in IL34-KO cultures relative to WT cultures (Figure S6L and M). After one day in differentiation medium, the ratio of MyoD^-^Ki67^+^ to MyoD^+^Ki67^+^ cells was evidently higher in IL34-KO cultures than in WT cultures (Figure 2K and L). The number of Pax7-positive cells beneath laminin was dramatically higher on transverse sections of TA muscle at 14 days postinjury from IL34-KO mice than on those from WT mice (Figures 2M, N and S5C, D). As inactivation of IL34 leads to the enhanced Pax7^+^ cell maintenance in the process of skeletal muscle regeneration, we then induced skeletal muscle damage in a second round by injecting 1.2% BaCl_2_ into the TA muscle and allowing a recovery period of 5 days. Interestingly, the regenerative capability of IL34-KO mice was significantly increased at early acute injury stage relative to control groups (Figure 2O and P). IL34 mainly secreted by myocytes and regenerating myofibers is essential for inducing the differentiation of self-renewed Pax7^+^ cell to further fuse into regenerating myofibers to fulfill muscle repair. We then explored myonuclear domain in the process of skeletal regeneration between Ctrl and *IL34^CKO^* mice. Analysis of nuclei number in isolated EDL myofibers 14 days after 1.2% BaCl_2_ treatment revealed that *IL34^CKO^* mice displayed increase in the myonuclear domain (Figure 2Q and U). These results suggest that mice without IL34 show increased expansion of the satellite stem cell pool via reduced differentiation of SCs in the process of muscle repair.

### Losing IL34 increases the transcription of Igfbp5 during myogenesis

**Figure 3:**
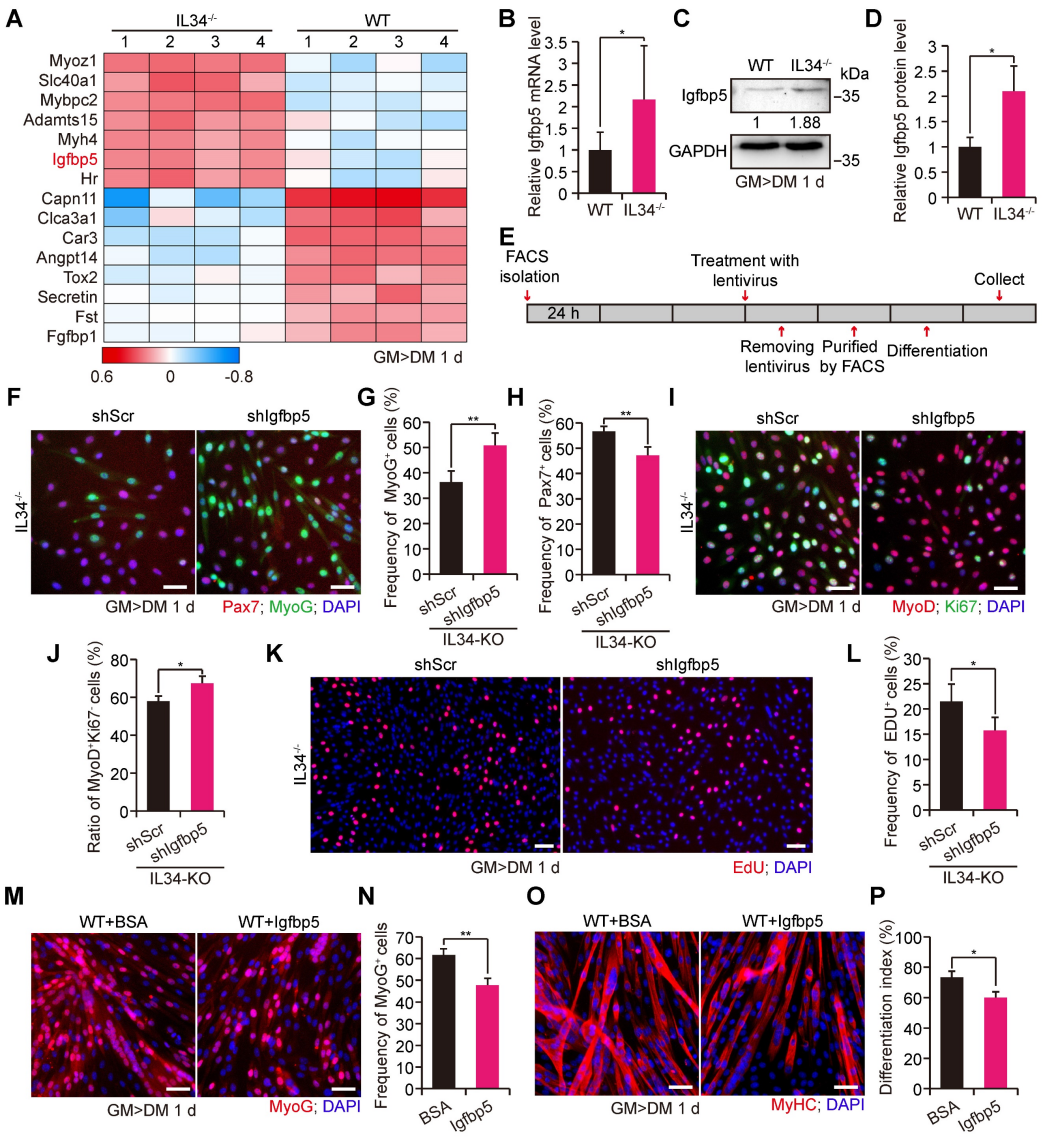
**IL34 represses the transcription of Igfbp5 during muscle repair. (A)** Heatmaps of genes with up- and downregulated expression in differentiated SCs after inactivation of IL34.** (B)** Relative Igfbp5 mRNA levels in day 1 differentiated WT and IL34-KO cultures detected using RT-qPCR. *p<0.05.** (C)** Western blot analysis of Igfbp5 and unrelated β-tubulin in day 1 differentiated WT and IL34-KO cultures. **(D)** Relative Igfbp5 protein levels in day 1 differentiated WT and IL34-KO cultures. *p<0.05.** (E)** Experimental procedure outlining the use of stable shIgfbp5 and shScr lentivirus-infected IL34-KO SCs for cell fate determination analysis. **(F)** Representative merged images of stably infected IL34-KO SCs stained for Pax7, MyoG and DAPI. Scale bar: 30 μm. **(G and H)** Quantitative analysis of the frequency of MyoG^+^ **(G)** and Pax7^+^** (H)** cells in shScr- and shIgfbp5-infected IL34-KO cultures. **p<0.01. **(I)** Representative immunofluorescence analysis of MyoD, Ki67 and DAPI costaining in shScr- and shIgfbp5-infected IL34-KO cultures. Scale bar: 30 μm. **(J)** Quantification of the percentage of differentiated MyoD^+^Ki67^-^ cells from shScr- and shIgfbp lentivirus-treated IL34-lacking SCs. *p<0.05. **(K)** Representative merged photomicrographs of stably infected IL34-KO SCs stained with EdU and DAPI. Scale bar: 50 μm.** (L)** Proportion of EdU^+^ cells in shIgfbp5- and shScr-treated IL34-KO SCs. *p<0.05. **(M)** Primary myoblasts were established from the hind limbs of WT mice and then expanded to induce differentiation for 1 day in medium containing BSA or Igfbp5 recombinant when cells were 70% confluent. Representative immunofluorescence analysis of MyoG and DAPI costaining in BSA- and recombinant Igfbp5-treated WT cultures. Scale bar: 30 μm. **(N)** Quantification of the percentage of differentiated MyoG cells from BSA- and recombinant Igfbp5-treated WT cultures. **p<0.01. **(O)** Primary WT myoblasts were cultured in differentiation medium containing BSA or Igfbp5 recombinant for 1 day. Myogenic differentiation was determined by immunostaining for MyHC. Scale bar: 30 μm. **(P)** Measurement of the differentiation index was determined by the frequency of nuclei in MyHC^+^ cells. *p<0.05.

From the significant differential gene heatmap analysis, we focused on insulin-like growth factor binding protein-5 (Igfbp5), which is markedly expressed in differentiating SCs from IL34-KO mice (Figure 3A). The dramatically enhanced transcription level of Igfbp5 in differentiating IL34-KO SCs was further confirmed via mRNA and protein level detection (Figure 3B-D).

To clarify whether IL34 regulated skeletal regeneration mainly through effects on Igfbp5 functions, we used a lentivirus expressing shRNA that specifically targeted Igfbp5 (shIgfbp5) to examine cell fate determination (Figure 3E). shIgfbp5 treatment in IL34-KO cultures led to dramatically enhanced differentiation, as shown by a significant increase in differentiated MyoG cells and MyoD^+^Ki67^-^ (Figure 3F, G and 3I, J) and a reduction in Pax7 cells (Figure 3F and 3H). The percentage of EdU-labeled proliferating cells in shIgfbp5-treated IL34-KO differentiation cultures was clearly decreased relative to that in shScr-treated groups (Figure 3K and L). We also augmented Igfbp5 function by adding recombinant Igfbp5 to WT differentiating cultures, and Igfbp5 treatment drastically blunted myogenic lineage progression, as indicated by decreases in the number of MyoG cells and the differentiation index in WT cultures treated with Igfbp5 compared to BSA-treated cultures (Figure 3M-P). Thus, we conclude that enhanced Igfbp5 function caused by inactivation of IL34 plays a pivotal role in improving Pax7^+^ cell maintenance by reducing differentiation.

### PI3K-AKT signaling acts as an effective downstream target of the IL34-Igfbp5 axis to modulate myogenesis

**Figure 4:**
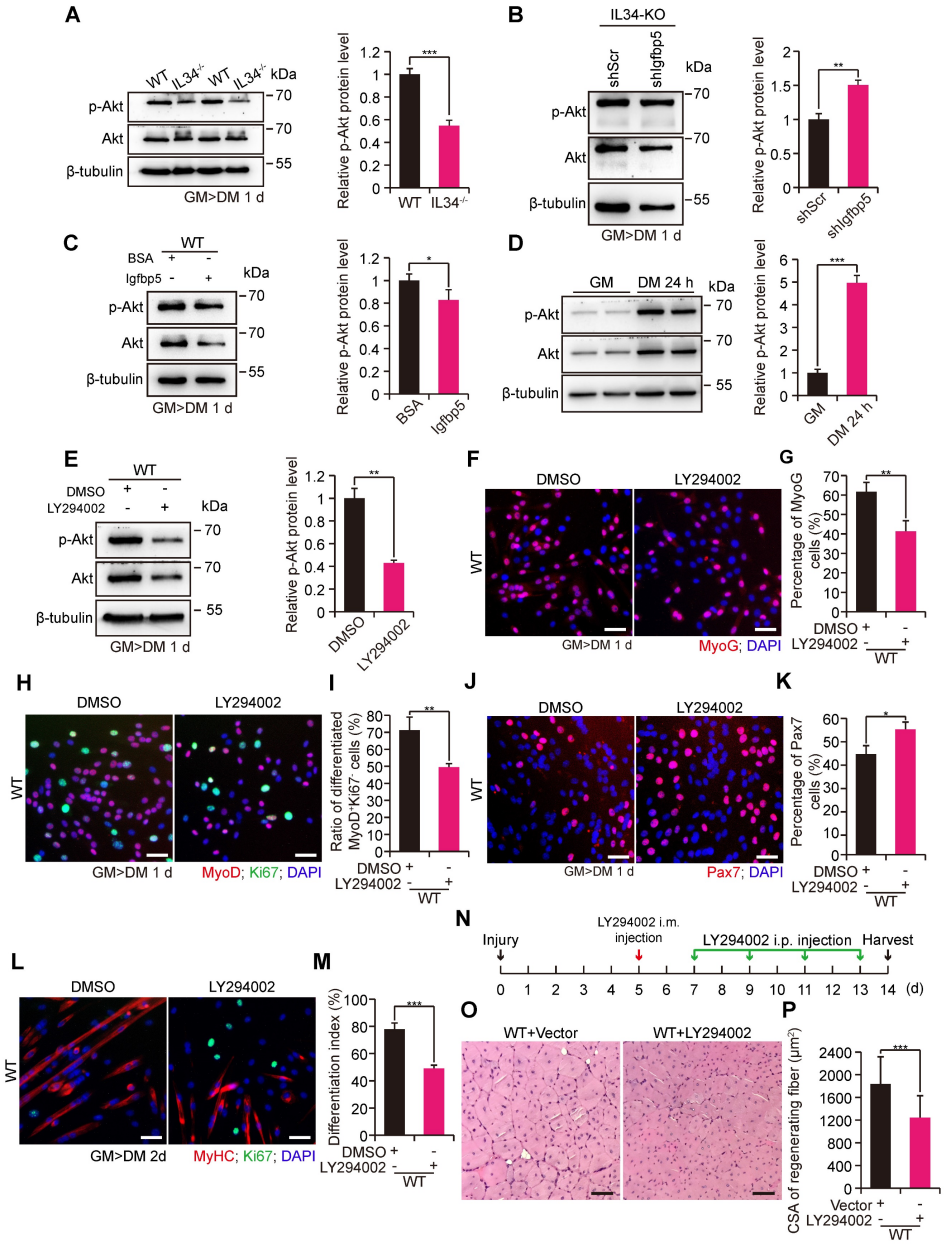
** IL34-Igfbp5 axis-mediated SC function through upregulation of PI3K-AKT activity. (A)** Left panel, immunoblot analysis of relative protein levels of p-AKT, total AKT and unrelated β-tubulin in 1-day differentiated WT and IL34-KO cultures. Right panel, relative p-Akt protein levels in 1-day differentiated WT and IL34-KO cultures. ***p<0.001.** (B)** Left panel, western blot analysis of p-AKT, total AKT, and unrelated β-tubulin protein levels in 1-day differentiated IL34-depleted SCs stably infected with shScr or shIgfbp5 lentivirus. Right panel, relative p-Akt protein levels in 1-day differentiated IL34-depleted SCs stably infected with shScr or shIgfbp5 lentivirus. **p<0.01. **(C)** Left panel, western blot analysis of p-AKT, total AKT, and unrelated β-tubulin protein levels in 1-day differentiated WT SCs treated with BSA or recombinant Igfbp5. Right panel, relative p-Akt protein levels in 1-day differentiated WT SCs treated with BSA or recombinant Igfbp5. *p<0.05.** (D)** Left panel, western blot analysis of p-AKT, AKT and unrelated β-tubulin protein levels in proliferating myoblasts and 1-day differentiated myoblasts from WT mice. Right panel, relative p-Akt protein levels in proliferating myoblasts and 1-day differentiated myoblasts from WT mice. ***p<0.001. **(E)** Left panel, immunoblot analysis of the relative protein levels of p-AKT, total AKT and unrelated β-tubulin in 1-day differentiated WT SCs treated with DMSO or LY294002 for 24 h. Right panel, relative p-Akt protein levels in proliferating myoblasts and 1-day differentiated WT SCs treated with DMSO or LY294002 for 24 h. **p<0.01. **(F)** FACS-isolated SCs were first plated in growth medium to expand and then switched to differentiation medium plus DMSO or LY294002 when cells were 70% confluent. Representative immunofluorescence analysis of MyoG and DAPI costaining in WT SC cultures treated with DMSO or LY294002. Scale bar: 30 μm. **(G)** Quantification of the percentage of differentiated MyoG cells from differentiated WT SCs treated with DMSO or LY294002. **p<0.01. **(H)** Representative overlapping images of DMSO- or LY294002-treated differentiated WT SCs stained against MyoD, Ki67 and DAPI. Scale bar: 30 μm. **(I)** Quantification of the frequency of MyoD^+^Ki67^-^ cells from WT SCs in differentiation medium supplemented with DMSO or LY294002. **p<0.01. **(J)** Representative merged photomicrographs of Pax7-stained WT SCs cultured in differentiation medium containing DMSO or LY294002. Nuclei were labeled with DAPI. Scale bar: 30 μm. **(K)** Frequency of undifferentiated Pax7^+^ cells in WT SCs cultured in differentiation medium containing DMSO or LY294002. *p<0.05. **(L)** Representative immunofluorescence analysis of WT SCs cultured in differentiation medium containing DMSO or LY294002. MyHC and Ki67 were stained to visualize the differentiation index, and DNA was stained with DAPI. Scale bar: 30 μm. **(M)** Measurement of the differentiation index detected by calculating the percentage of nuclei in MyHC-positive cells. ***p<0.001.** (N)** LY294002 hindered AKT activity in skeletal muscle regeneration. The beginning of LY294002 treatment was on the 5^th^ day after injury via intramuscular injection, the later injection was performed every 2 days via intraperitoneal injection, and samples were collected 1 day after the fifth LY294002 treatment.** (O)** Representative H&E-stained transverse sections of TA muscle from WT mice treated with vector or LY294002 at 14 days postinjury. Scale bar: 50 μm. **(P)** Quantification of the average CSA of myofibers with centralized nuclei. ***p<0.001.

As Igfbp5 binding to IGFs gives rise to effective inhibition of IGF signaling by disrupting IGF binding to IGFRs, we speculated that PI3K-AKT signaling might be involved in the skeletal muscle regeneration process via the IL34-Igfbp5 axis. The level of phosphorylated AKT protein was dramatically decreased in the IL34-KO cultures compared to those in the WT cultures (Figure 4A). We then infected primary myoblasts isolated from adult IL34-KO mice with a lentivirus expressing shIgfbp5 or a control shRNA (shScr), and stably infected cells were purified by performing a FACS-based assessment. After one day in differentiation medium, we observed that shIgfbp5 treatment caused a significant increase in p-AKT protein levels (Figure 4B). We also measured AKT activity by adding mouse recombinant Igfbp5 to WT cultures and observed an evident decrease in p-AKT protein levels (Figure 4C). These results demonstrate that Igfbp5 functions as a negative modulator of PI3K-AKT signaling during skeletal muscle regeneration.

We examined the extent to which phosphorylated AKT levels were strongly increased in WT myoblast cultures after induction of differentiation for one day relative to proliferating myoblasts (Figure 4D). We next set out to interfere with PI3K-AKT signaling by treating WT myoblasts subjected to differentiation culture with a commonly used PI3K inhibitor, LY294002, for 24 h. LY294002 treatment effectively blocked PI3K-AKT signaling, as observed by a clear decrease in p-AKT protein levels (Figure 4E). Treatment with LY294002 led to a marked decrease in the numbers of both MyoG and MyoD^+^Ki67^-^ cells (Figure 4F-I). Alternatively, there was a persistently high percentage of Pax7 cells (Figure 4J and K). We also illustrated that inhibition of PI3K-AKT caused a failure to initiate terminal differentiation, as shown by a lower differentiation index than that yielded by treatment with the DMSO control (Figure 4L and M). In addition, we injected LY294002 into the regenerating TA muscle of adult WT mice to investigate the recovery of damaged muscle. Interestingly, we observed that utilization of LY294002 at the beginning of day five after injury resulted in a dramatic reduction in the average myofiber size when regeneration occurred on the 14^th^ day postinjury (Figure 4N-P). These results revealed that PI3K-AKT signaling is essential for myogenic lineage progression.

### IL34 inactivation-mediated augmented Igfbp5 transcription is dependent on NFKB1 signaling

**Figure 5:**
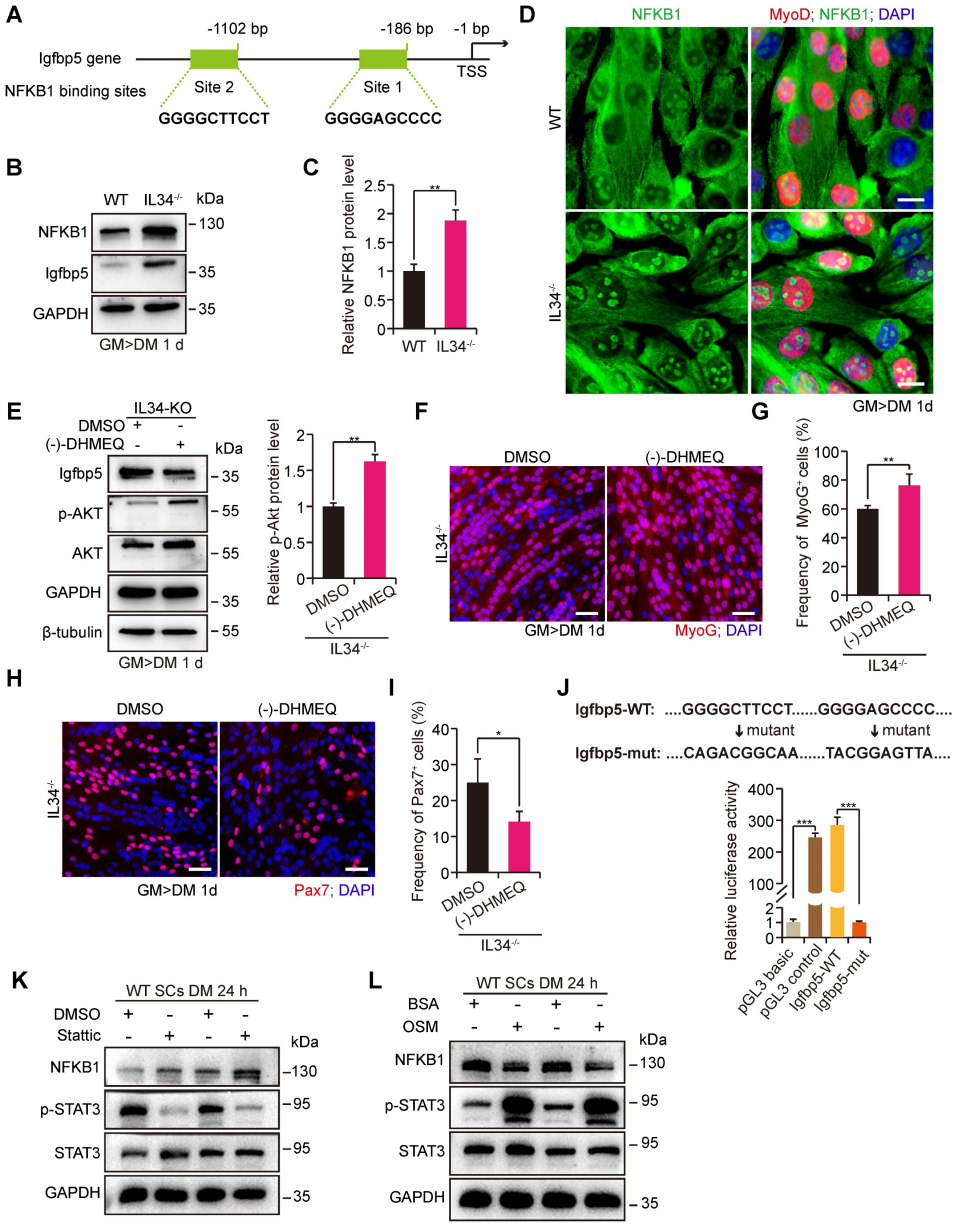
** IL34 inactivation resulting in overactivated NFKB1 activity upregulates Igfbp5 transcription. (A)** Schematic diagram presenting 2 potential NFKB1 binding sites in the Igfbp5 promoter. **(B)** Western blot analysis of NFKB1, Igfbp5 and unrelated GAPDH protein levels in 1-day differentiated WT and IL34-KO cultures.** (C)** Relative NFKB1 protein levels in 1-day differentiated WT and IL34-KO cultures. **p<0.01. **(D)** Representative immunofluorescence analysis of WT and IL34-KO primary myoblasts cultured in differentiation medium for 1 day. NFKB1 and MyoD were stained to visualize the nuclear localization signal of NFKB1, and DNA was stained with DAPI. Scale bar: 20 μm. **(E)** Left panel, immunoblot analysis of the protein levels of Igfbp5, p-AKT, total AKT and unrelated β-tubulin and GAPDH in 1-day differentiated IL34-KO cultures treated with DMSO or (-)-DHMEQ. Right panel, relative p-Akt protein levels in 1-day differentiated IL34-KO cultures treated with DMSO or (-)-DHMEQ. **p<0.01. **(F)** Representative overlapping images of 1-day differentiated IL34-KO SCs treated with DMSO or (-)-DHMEQ after labeling with MyoG and DAPI. Scale bar: 30 μm. **(G)** Quantification estimation of the ratio of MyoG^+^ cells in 1-day differentiated IL34-KO SCs treated with DMSO or (-)-DHMEQ. **p<0.01. **(H)** Representative merged photomicrographs of 1-day differentiated IL34-KO SCs treated with DMSO or (-)-DHMEQ after staining with Pax7 and DAPI. Scale bar: 30 μm.** (I)** Percentage of Pax7^+^ cells in differentiated IL34-KO cultures treated with DMSO or (-)-DHMEQ. *p<0.05. **(J)** Relative luciferase activity in C2C12 cells electrotransfected with phRL-TK plasmid and pGL3-basic empty vector (pGL3 basic), pGL3-control vector (pGL3 control), wild-type Igfbp5 promoter (Igfbp5-WT), or mutant Igfbp5 promoter (Igfbp5-mut) with mutation of NFKB1 binding sites under normal conditions. ***p<0.001. **(K)** Isolated WT myoblasts from the adult mice were initially cultured in growth medium then induced to differentiation by switching growth medium into differentiation medium with or without Stattic. Western blot analysis of the levels of NFKB1, p-STAT3, STAT3 and an unrelated protein (GAPDH) in WT myoblasts grown in differentiation medium after the addition of 2 μM Stattic. **(L)** Isolated WT myoblasts from the adult mice were initially undergone expansion in growth medium and then induced to differentiation in medium with or without OSM. Western blot analysis of the levels of NFKB1, p-STAT3, STAT3 and an unrelated protein (GAPDH) in SCs grown in differentiation medium containing 50 ng/ml OSM.

Loss of IL34 function in differentiating SCs caused the forced transcription of Igfbp5, which perturbed cell fate determination by reducing PI3K-AKT signaling activity. We wondered about the transcriptional regulatory mechanisms by which IL34 controls Igfbp5 expression. Scanning of the promoter region in the 2-kb region upstream from the TSS of Igfbp5 by employing the JASPAR database revealed that NFKB1 could be an essential transcription factor for Igfbp5, in the promoter area of which 2 binding sites were analyzed (Figure 5A). We next detected significant hyperactivation of NFKB1 signaling in IL34-KO SC cultures compared to the WT cultures at day one after induction of differentiation (Figure 5B-D). Intriguingly, treating IL34-KO cultures with commonly used chemical reagent inhibitors of NF-κB signaling, (-)-DHMEQ, resulted in a remarkable reduction in Igfbp5 expression levels and an increase in Akt activity (Figure 5E). We also showed that MyoG cells were evidently augmented in differentiating IL34-KO cultures treated with NF-κB inhibitors (Figure 5F and G). The number of Pax7 cells decreased in the differentiating IL34-KO cultures treated with NF-κB inhibitors (Figure 5H and I).

To further verify the regulatory effect of NFKB1 on Igfbp5 transcription, the Igfbp5 promoter region and variants with mutations in NFKB1 binding sites were cloned into a luciferase reporter. Augmented Igfbp5 promoter activity was detected with wild-type NFKB1-binding sites, while this activity was abolished when NFKB1-binding sites were mutated in the Igfbp5-promoter region (Figure 5J). We manipulated the STAT3 activity in cultured WT SCs could lead to the NFKB1 activity changes in a negative regulation manner (Figure 5K and L), suggesting that IL34-controled STAT3 activity is a causal relation between IL34 and NFKB1 13. These results demonstrate that hyperactivation of NFKB1 activity in IL34-KO cultures directly induces Igfbp5 transcription.

### Genetic inactivation of IL34 improves the dystrophic phenotype in mdx mice

**Figure 6:**
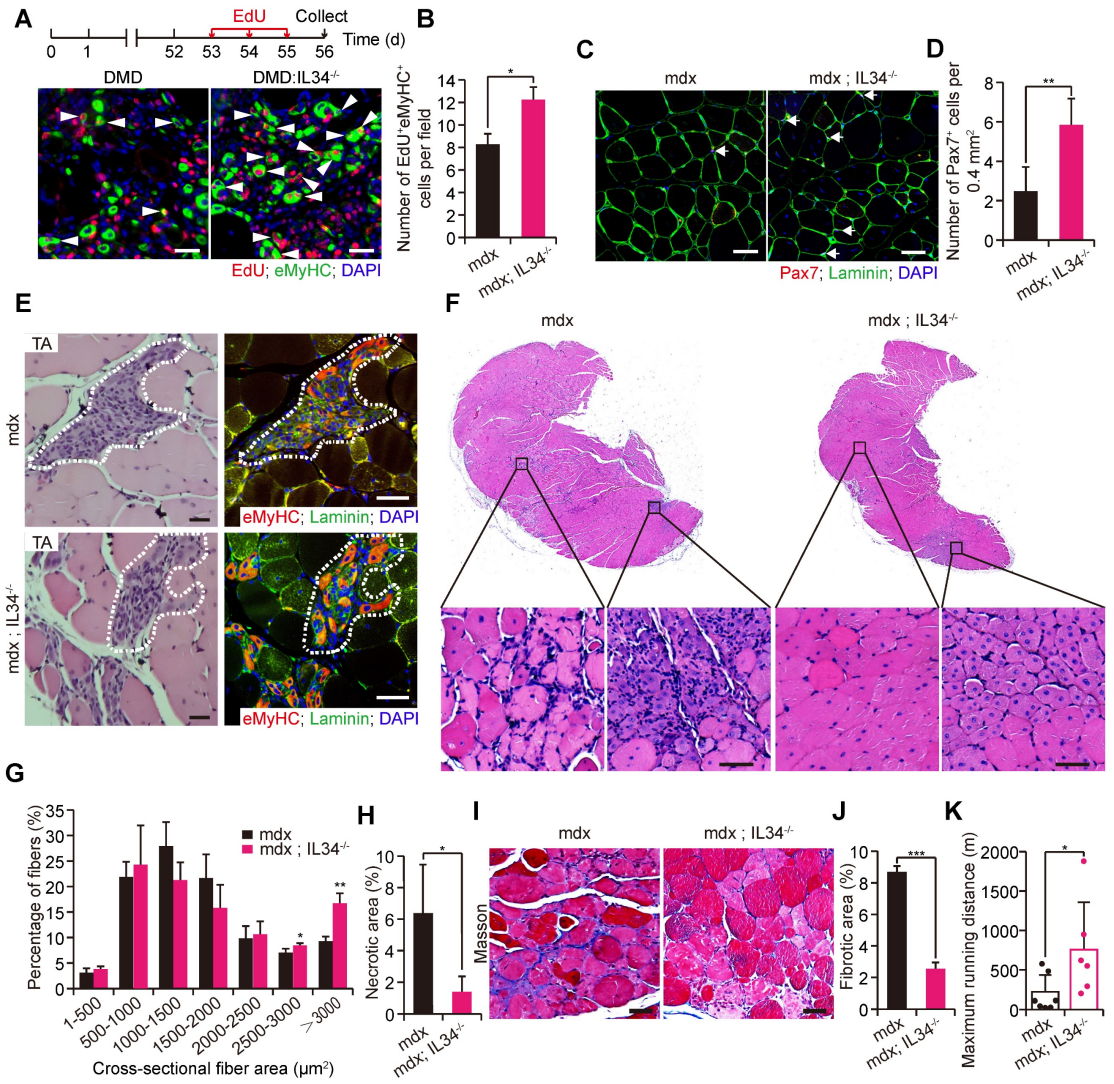
** IL34 inactivation attenuates Duchenne muscular dystrophy in mouse models. (A)** Representative overlapping images of eMyHC^+^EdU^+^ cells on transverse sections of the Gas muscle from mdx and mdx::IL34^-/-^ mice at 8 weeks of age. Scale bar: 30 μm.** (B)** Quantification number of eMyHC^+^EdU^+^ cells per unit field. *p<0.05.** (C)** Representative merged images of Pax7 and laminin coimmunostained TA sections from mdx and mdx::IL34^-/-^ mice. Nuclei was labeled with DAPI. Scale bar: 30 μm.** (D)** Number of Pax7^+^ cells per field. **p<0.01.** (E)** Left, H&E-stained TA sections from mdx and mdx::IL34^-/-^ mice at 8 weeks of age; the marked area presents regenerating muscle fibers. Scale bar: 30 μm. Right, representative merged images of eMyHC and laminin coimmunostained serial TA muscle sections. The same marked area presents eMyHC-positive regenerating muscle fibers. Scale bar: 50 μm.** (F)** H&E staining analysis of transverse sections of Gas muscle from mdx and mdx::IL34^-/-^ mice. Scale bar: 50 μm.** (G)** Quantification of regenerating fiber size (frequency) distribution of mdx and mdx::IL34^-/-^ mice. N=4 mice in each group. *p<0.05 and **p<0.01.** (H)** Quantification of necrotic area in Gas muscle *p<0.05.** (I)** Masson's staining analysis of fibrosis accumulation in transverse sections of Gas muscle from mdx and mdx::IL34^-/-^ mice. Scale bar: 50 μm.** (J)** Measurement of the fibrotic area. ***p<0.001.** (K)** Running distance to exhaustion on the downhill treadmill test. *p<0.05.

As gradual exhaustion of the satellite stem cell pool is an obvious pathological outcome among patients who suffer from the extremely severe muscle disease Duchenne muscular dystrophy, the positive effect of IL34 inactivation on satellite stem cell pool maintenance during both in vivo and in vitro myogenesis led us to consider whether it might be helpful for ameliorating the muscular dystrophy phenotype in mdx mice. We generated mdx mice lacking IL34 (mdx::IL34^-/-^) and compared their muscular pathological phenotype with that of mdx mice. We administered EdU to eight-week-old mice via consecutive intraperitoneal injection, which allowed us to track cells that entered the cell cycle to repair damaged skeletal muscle during this period, and we found that a lack of IL34 evidently promoted the expansion of myogenic cells, as shown by increased EdU incorporation into eMyHC^+^ cells 3 d after injection (Figure 6A and B). A higher number of Pax7-positive cells was detected in transverse sections of the TA muscle from mdx::IL34^-/-^ mice than in those of the TA muscle from mdx mice (Figure 6C and D). Moreover, compared with that in mdx mice, the number of developmental myosin heavy chain (eMyHC)-positive myofibers was also dramatically increased in both the TA and gastrocnemius (Gas) muscles of mdx::IL34^-/-^ mice (Figures 6E and S7A, B), and the frequency of eMyHC-positive myofibers containing 2 or more than 2 nuclei was also dramatically increased in the muscles of mdx::IL34^-/-^ mice (Figure S7C).

H&E staining of Gas transverse sections revealed that there were markedly larger fibers, fewer infiltrating cells and fewer necrotic areas in 24-week-old mdx::IL34^-/-^ mice than in mdx mice (Figure 6F-H). Additionally, analysis of fibrosis accumulation revealed that the content of fibrous scar tissue was also decreased by deleting IL34 in mdx mice (Figure 6I and J). At 24 weeks of age, mdx::IL34^-/-^ mice ran for a significantly longer distance than mdx mice, indicating that they had less muscle injury and less dysfunction (Figure 6K).

### Interference with PI3K-AKT signaling in vivo ameliorates dystrophic myopathy in mdx mice

**Figure 7:**
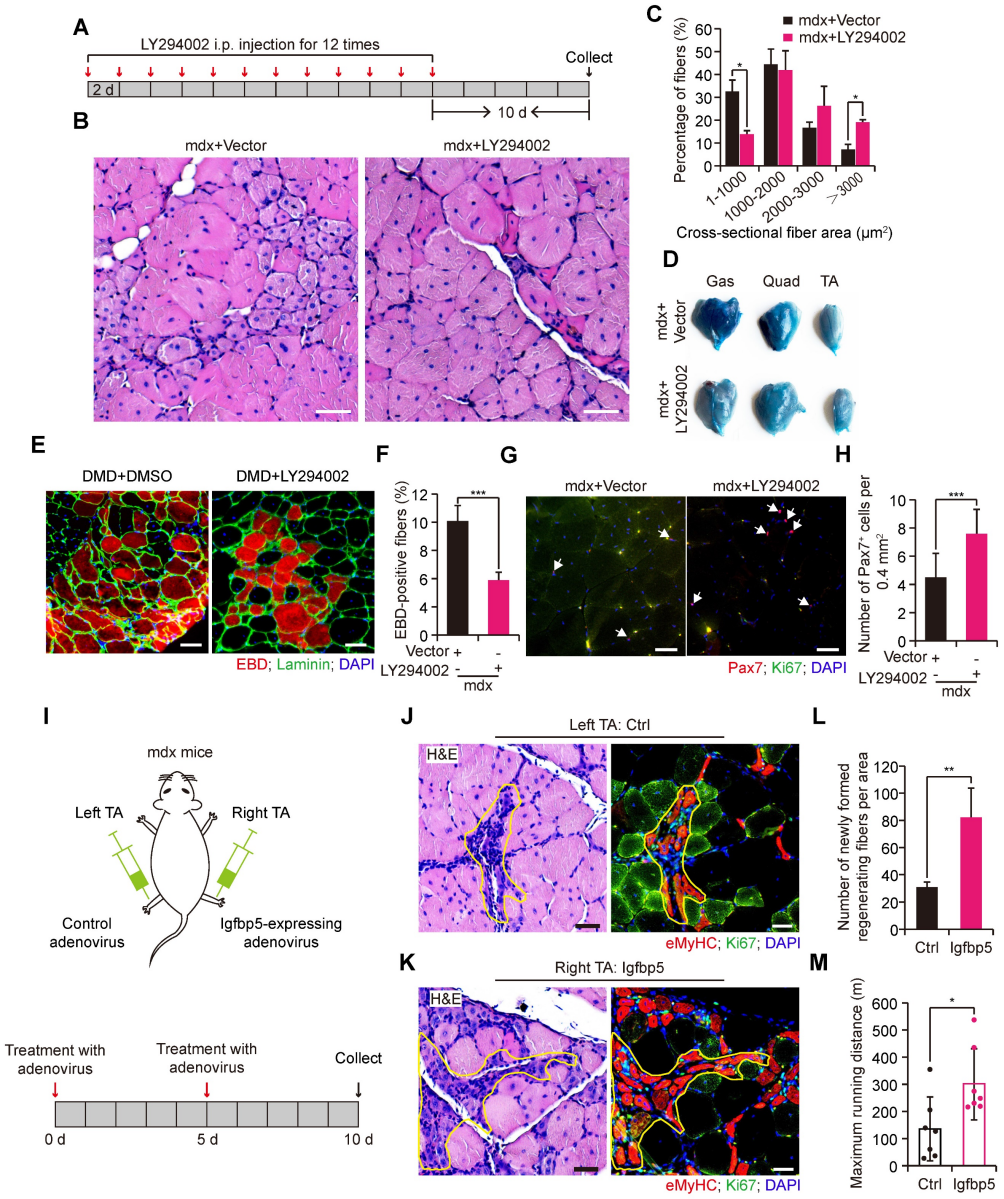
** PI3K-AKT signaling inhibition in vivo improves muscular dystrophy in mdx mice. (A)** Experimental procedure presenting the utilization of LY294002 in wild-type mdx mice for skeletal muscle pathological performance analysis.** (B)** H&E staining analysis of transverse sections of Gas from wild-type mdx mice treated with vector or LY294002 (5 mg/kg per i.p. injection). Scale bar: 50 μm.** (C)** Measurement of fiber size distribution of nuclei-centralized myofibers of Gas muscle from wild-type mdx that received repeated intraperitoneal injection of LY294002 for 12 times. N=6 mice in each group. *p<0.05.** (D)** Results of Evans blue dye (EBD) uptake of Gas, Quad and TA muscle of mdx mice treated with vector or LY294002. **(E)** Evans blue dye uptake in TA muscle of mdx mice treated with vector or LY294002. Scale bar: 50 μm. **(F)** Frequency of EBD positive fibers. ***p<0.001. **(G)** Representative merged images of Pax7 and Ki67 coimmunostained TA sections from WT mdx mice treated with vector or LY294002. Nuclei was labeled with DAPI. Scale bar: 30 μm.** (H)** Number of Pax7^+^ cells per field. ***p<0.001.** (I)** Experimental schematic outlining the treatment of wild-type male mdx mice at 2 months of age with control adenovirus or Igfbp5-expressing adenovirus.** (J)** Left, H&E-stained transverse section of TA muscle treated with control adenovirus; the marked area presents regenerating muscle fibers. Scale bar: 30 μm. Right, representative merged images of eMyHC and Ki67 coimmunostained serial TA muscle sections. The same marked area presents eMyHC-positive regenerating muscle fibers. Scale bar: 50 μm.** (K)** Effect of Igfbp5 overexpression on the histology of TA muscle isolated from 2-month-old mdx mice. Left, H&E-stained transverse section of TA muscle treated with Igfbp5-expressing adenovirus; the marked area presents regenerating muscle fibers. Scale bar: 30 μm. Right, representative merged images of eMyHC and Ki67 coimmunostained serial TA muscle sections. The same marked area presents eMyHC-positive regenerating muscle fibers. Scale bar: 50 μm.** (L)** Number of newly formed regenerated myofibers in TA muscle that received intramuscular control adenovirus and Igfbp5-expressing adenovirus injection. N=3 male mice. **p<0.01. **(M)** Limb muscles of 4-months old mdx mice were intramuscularly given Igfbp5-expressing adenovirus. Running distance to exhaustion on the downhill treadmill test. *p<0.05.

The positive effect of IL34 deletion on regeneration in the background of mdx mice led us to consider whether AKT inhibition might also be helpful for improving dystrophic muscles in mdx mice. For this, we treated adult mdx mice with repeated intraperitoneal (IP) injections of LY294002 to measure the muscular pathological performance (Figure 7A). We observed that Akt activity inhibition resulted in better muscle performance, as indicated by markedly larger fibers in 2-month-old mdx mice treated with LY294002 (Figure 7B and C). Furthermore, myofiber membrane permeability, measured by Evans blue dye (EBD) uptake, was also alleviated in mdx mice after i.p. injection of PI3K-AKT inhibitor (Figure 7D-F). There was a significantly higher number of Pax7^+^ cells in the transverse sections of Gas muscle from mdx mice that received LY294002 injection (Figure 7G and H). To test whether overexpression of Igfbp5 in the skeletal muscle of mdx mice could improve muscle performance, we administered Igfbp5-expressing adenovirus into the right TA muscle of adult mdx mice and control adenovirus into the left TA muscle of adult mdx mice (Figure 7I). We observed remarkably increased newly formed regenerating myofibers in Igfbp5-expressing adenovirus-treated TA muscles (Figure 7J-L). Mdx mice injected Igfbp5-expressing adenovirus into limb muscles ran for a significantly longer distance than mdx mice treated with control adenovirus (Figure 7M).

## Discussion

Because IL34 deletion causes the proliferation and self-renewal of Pax7 cells by sacrificing MyoG cells, reduces SC exhaustion and increases the number of Pax7 cells in dystrophic muscle, it is helpful for improving dystrophic muscular performance. Reports have illustrated that IL34 functions as a secreted cytokine 28, the transcription level of which is enriched in differentiated myocytes and newly formed myotubes, suggesting that myocytes and myotubes could direct Pax7^+^ cells to initiate differentiation by releasing IL34 to the Pax7^+^ cell environment and further indicating that IL34 is an essential regulator that is responsible for inducing renewed Pax7^+^ cells to further differentiate to completely repair damaged fibers.

Inactivation of canonical NF-κB signaling has been implicated in attenuating skeletal muscle regeneration by causing precocious differentiation of SCs 29, 30. Our results revealed that NFKB1 signaling was hyperactivated in IL34-inactivated SCs, the fates of which are inclined to expand but not differentiate, consistent with previous descriptions and further suggesting the importance of NF-κB signaling in myogenesis. Many reports have demonstrated that both IL34 and CSF1 can bind to CSF1R to give rise to similar or distinct biological activities but not redundant roles 28, 31-34. We also detected that inhibition of CSF1R via utilizing a commonly used CSF1R inhibitor, PLX5622, can clearly attenuate myogenic differentiation (Data not shown), indicating there is a possibility that loss of IL34 makes CSF1 release its potential abilities, thus triggering downstream reactions leading to NF-κB hyperactivation, as CSF1 has been demonstrated to regulate the development and homeostasis of macrophages by upregulating NF-κB activity 35.

Hyperactivated NFKB1 activity in IL34-KO SCs cause enhanced transcription of Igfbp5, which is secreted into the extracellular space and cooperates with environmental IGFs with high affinity 36, 37. Igfbp5 is one of the targets of miR-206, the loss of function of which hampers myogenic lineage progression 38. Although direct descriptions of Igfbp5 overexpression in the regulation of muscle regeneration are lacking, our results are consistent with inactivation of miR-206-induced SC differentiation defects. Exercise upregulates the expression of Igfbp7, which impedes the activity of the Akt-mTOR axis to protect Pax7 cell maintenance 17. We deduced that distinct Igfbp family members may share functional properties in regulating the fate determination of SCs under different physiological and pathological conditions.

PI3K-AKT cooperation with mTOR signaling plays an essential role in the cell cycle reentry of quiescent SCs upon exposure to extrinsic stimuli 39. Our results initially uncovered the significance of PI3K-AKT in promoting the eventual differentiation of SCs during muscle regeneration. Although pharmacologically treating regenerating muscle with LY294002 results in regeneration deficiency due to differentiation deficiency of SCs, consecutive repeated utilization of LY294002 in severe muscular dystrophy disease decreases SC exhaustion, thus ultimately attenuating Duchenne muscular dystrophy in mouse models. The effect of inhibition of Akt activity on muscle regenerative capability is extremely similar to that of depletion of IL34. Future studies will further elucidate the disruption of PI3K-AKT signaling as a therapeutic modality for improving muscle mass and function in DMD.

## Materials and Methods

### Animals

The IL34^-/-^ and IL34-floxed mice used for this research project were established on a C57BL/6 background. The design and generation of these mice were authorized to a commercial organization. F1 generation adult mice with IL34^+/-^ and IL34^+/flox^ genotypes were delivered to us to produce mice on the available genetic backgrounds, and all the experimental processes were strictly performed according to the standard operating protocols of Cyagen Biosciences, Inc. *Pax7^CreER^* and mdx mice have been described previously 40. All mice were housed under sterile conditions and had access to food and water. IL34^+/-^ mice were initially bred with WT C57BL/6 mice to produce F2 generation IL34^+/-^ mice, which were intercrossed to generate IL34^-/-^ mice. IL34^+/+^ littermate mice were used as controls. IL34^+/flox^ mice were intercrossed to generate IL34^flox/flox^ mice. Pax7^CreER^ mice and IL34^flox/flox^ mice were used to generate Pax7^CreER^:IL34^flox/flox^ mice, and IL34^flox/flox^ or IL34^+/flox^ littermates were used as controls. Tamoxifen (Sigma-Aldrich) injection for Cre recombinase activation was performed as previously described 41. All animal protocols performed in this study were approved by the Regulations of Beijing Laboratory Animal Management and strictly obeyed the guidelines of the China Agricultural University Laboratory Animal Welfare and Animal Experimental Ethical Inspection Form (approval number: AW92609102-3-1).

### Myofiber isolation, culture and nuclei count

Myofiber isolation and culture were performed as described previously 13. Briefly, EDL muscle was carefully isolated from adult IL34-KO, IL34^CKO^, Ctrl and WT mice and digested with collagenase I (Sigma-Aldrich, C0130). The dispersed single myofibers were transferred under a microscope and then suspended in a 48-well plate precoated with horse serum. Isolated myofibers were incubated in DMEM supplemented with 10% horse serum, 0.5% chick embryo extract, and 1% penicillin-streptomycin. The fibers were cultured for 24 h, 48 h or 72 h at 37°C in a 5% CO_2_ atmosphere. Freshly isolated fibers were fixed with 4% paraformaldehyde (PAF) in PBS for 20 min and stained for Pax7, Ki67 and IL34.

To measure nuclei count on single myofibers isolated from day 14 injured skeletal muscle of Ctrl and IL34^CKO^ mice, dispersed single myofibers from EDL muscle were immediately fixed with 4% paraformaldehyde (PAF) in PBS for 20 min and stained for DAPI. Total number of myonuclear was determined by using ImageJ Software. Six spots along the length of the fiber were performed to measure the average diameter of the fiber. Using the average diameter and length of the fiber to calculate the volume of the fiber. Myonuclear domain was acquired by using the volume of the fiber to divide the myonuclear number of the fiber.

### Cell storage

Purified SC isolation was performed by FACS as described previously 13. Briefly, the hind limb skeletal muscle of adult mice was carefully isolated, minced and digested to yield mononuclear cell suspensions. Mononuclear cells were stained with α-integrin-7-APC (clone; R2F2; Ablabs), CD31-PE/Cy7 (clone 390; Biolegend), Sca1-PerCp (clone D7; eBioscience) and CD45-FITC (clone 30-F11; BD Bioscience) antibodies in cold PBS supplemented with 3% fetal bovine serum for 1 h. A Beckman cell sorter equipped with lasers was used for cell storage. Cells stained with α-integrin-7-APC and without CD45-FITC, CD31-PE/Cy7 and Sca1-PerCp were enriched for SCs. Stored SCs were initially maintained in DMEM supplemented with 20% FBS, 10 ng/ml bFGF, 1% chick embryo extract, and 1% penicillin-streptomycin at 37°C in a 5% CO_2_ atmosphere. Cells reached 70% confluency, and the growth medium was switched to differentiation medium containing DMEM, 5% horse serum and 1% penicillin-streptomycin.

### Primary myoblast isolation

The hind limb skeletal muscle of mice at 6-8 weeks was isolated and minced under sterile conditions, and then a mixture including collagenase II (500 units, Sigma-Aldrich, C6885) and dispase II (1.1 units per ml; Roche) was added for digestion at 37°C on a horizontal rocking bed for 45 min. Cells were filtered through a 100-μm nylon cell strainer, centrifuged and cultured in F-10 medium supplemented with 20% FBS, 10 ng/ml, 1% chick embryo extract, and 1% penicillin-streptomycin (myoblast growth medium) at 37°C in a 5% CO_2_ atmosphere. Supernatant mixed with cells was collected, centrifuged and maintained in myoblast growth medium at 37°C in 5% CO_2_. Cells at 70% confluency were induced to differentiate.

### Muscle injury and regeneration

To induce muscle regeneration, adult mice at 8-10 weeks of age were first anaesthetized by intraperitoneal injection of 20 mg/ml Avertin (Sigma-Aldrich) (15 μl/g). The right leg of each mouse was shaved and sterilized with 75% alcohol. Skeletal muscle regeneration was induced by injection of a total of 75 μL of 1.2% BaCl_2_ into the midbelly of the right TA muscle. After injection, mice were placed on a 37°C hot stage for recovery. Samples were harvested at 3, 5, 14 and 21 days after injury to assess the regenerative capability of the damaged muscle.

### Histological and morphometric analysis

Harvested muscle samples were initially fixed in 4% PFA at room temperature for 48 h and then subjected to routine paraffin histology. Transverse sections and longitudinal sections of skeletal muscle (5-μm thick) of the TA muscle were subjected to H&E staining. Quantification of the fiber cross-sectional area (CSA) was performed by using Aperio ImageScope software. More than 200 skeletal muscle fibers of each mice were measured, at least 3 mice in each group. Masson's trichrome was performed using a staining kit purchased from Solarbio according to standard procedures.

### Immunostaining of sections, cells and myofibers

After fixation, the cells and myofibers were washed three times with PBS; antigen retrieval was performed by heating muscle sections in Tris-EDTA buffer (pH 9.0), and the sections were then permeabilized in PBS containing 0.5% Triton X-100 (Sigma) and blocked in commercial blocking buffer (Beyotime, China) at room temperature. Cells, fibers or muscle sections were then incubated with primary antibody overnight at 4°C. Following incubation, all samples were briefly washed with PBS and incubated with secondary antibody at room temperature for one hour. DAPI staining was used to mark nuclei. The following primary antibodies were used: rabbit anti-IL34 (1:200, Abcam), mouse anti-Pax7 (1:100, Developmental Studies Hybridoma Bank (DSHB)), mouse anti-MyoG (1:200, Abcam), rabbit anti-MyoG (1:200, Santa Cruz), rabbit anti-Ki67 (1:500, Invitrogen), rabbit anti-laminin (1:1000, Sigma-Aldrich), mouse anti-MyHC (1:500, Sigma-Aldrich), mouse anti-eMyHC (1:40, DSHB), mouse anti-MyoD1 (1:100, DAKO), rabbit anti-NFKB1 (1:200, ABclonal).

### Construction and packaging of lentivirus and adenovirus

Construction and packaging of adenovirus and lentivirus granules were performed as previously described 13.

### Cell proliferation assay

For in vivo cell proliferation analysis, EdU at a dose of 20 mg/kg was intraperitoneally injected into mdx and mdx::IL34^-/-^ mice once a day for three days. Muscle samples were harvested one day after the last EdU injection.

For in vitro SC proliferation analysis, cells were labeled with EdU for the last 2 h using a Click-iT EdU Cell Proliferation Assay Kit (Invitrogen). Briefly, 2×10^4^ SCs were seeded on Matrigel-coated 24-well plates and incubated at 37°C in a 5% CO_2_ atmosphere for four days.

### Microarray and data analysis

For mRNA microarrays, total RNA samples were obtained from 4-day cultured proliferating SCs and 1-day differentiated SCs from WT and IL34-KO mice. Then, the RNA samples were submitted to Beijing igeneCode Biotech Co., Ltd. Library construction, sequencing and bioinformatics analysis were performed, strictly obeying corporate standard operation protocols.

### Western blotting

Total protein extracts were obtained by homogenizing cell cultures in RIPA buffer (Cell Signaling Technology, Cat: 9806) supplemented with protease inhibitor cocktail (Beyotime, China) and phosphatase inhibitors (Roche). Cell debris was removed by centrifugation, and the supernatant was collected and stored at -80°C. Protein concentration was measured using a BCA protein assay kit (Beyotime, China), and 30 μg of protein from different samples was then electrophoresed by 10% SDS-PAGE. Western blot analysis was performed using a standard protocol. The following antibodies were used: p-AKT (1:1000, CST), AKT (1:1000, CST), IL-34 (1:1000, Abcam), Igfbp5 (1:100, Scanta), NFKB1 (1:1000, ABclonal), p-STAT3 (1:1000, CST), STAT3 (1:1000, CST), MyHC (1:1000, Sigma-Aldrich), GAPDH (1:10000, CST), β-tubulin (1:10000, Abcam), horseradish peroxidase (HRP)-conjugated anti-mouse IgG (1:10000, CST), and HRP-conjugated anti-rabbit IgG (1:10000, CST).

### Treatment of SC cultures and damaged muscle with LY294002

To disrupt AKT activity in cultured SCs, SCs were initially expanded in growth medium and then induced to differentiate by switching growth medium to differentiation medium containing 10 μM LY294002 (Med Chem Express).

To explore the effects of AKT activity inhibition on skeletal muscle regeneration, we first intramuscularly injected LY294002 beginning five days after injury. Then, LY294002 (5 mg/kg) was intraperitoneally injected every two days for a total of four doses. Muscle samples were harvested one day after the last LY294002 injection. The vector used to deliver LY294002 to mice contained 10% DMSO, 40% PEG300, 5% Tween-80 and 45% saline. A vector containing the same volume of DMSO was also injected into the control mice.

To measure the effects of AKT activity inhibition on dystrophic muscular performance, we intraperitoneally injected LY294002 (5 mg/kg) into mdx mice at the age of two months. LY294002 injection was performed every two days for a total of twelve doses. Muscle samples were collected ten days after the last LY294002 injection. A vector containing the same volume of DMSO was also injected into the control mdx mice.

### In vitro inhibition of NF-κB activity

Primary myoblasts isolated from the hind limbs of IL34-KO mice were plated in 12-well culture dishes for expansion culture and then induced to differentiate in differentiation medium containing an NF-κB inhibitor: 10 μM (-)-DHMEQ (Med Chem Express). Cells cultured in differentiation medium containing the same volume of DMSO were used as control groups.

### In vivo overexpression of Igfbp5

To force the expression of Igfbp5 in the TA muscle of adult mdx mice, we treated injured muscle with an adenovirus expressing Igfbp5 or mCherry (control) in 0.9% NaCl, and adenovirus was injected at days 0 and 5. Muscle samples were collected at day 10. The viral dose was 6×10^11^ p.f.u. per damaged TA muscle. To measure maximum running distance, limb muscles of 4-months old mdx mice were treated with Igfbp5-expressing or mCherry-expressing adenovirus by intramuscular multiple injections. The viral dose was 6×10^12^ p.f.u. per mice.

### In vitro enhancement of Igfbp5 function and IL34 function

To force Igfbp5 function in cultured SCs, isolated SCs were first cultured in growth medium, and cells at 70% confluence were induced to differentiate by switching growth medium to differentiation medium containing 200 ng/ml recombinant Igfbp5 (Bio-techne); the same volume of 0.1% BSA was used as a control.

To force IL34 function in cultured SCs, isolated WT SCs and IL34-KO SCs were first cultured in growth medium, and cells at 70% confluence were induced to differentiate by switching growth medium to differentiation medium. After one day in differentiation medium, the medium was supplemented with 100 ng/ml recombinant IL34 (Origene); the same volume of 0.1% BSA was used as a control. After IL34 or BSA-treatment for 24 h, samples were collected for further analysis.

### In vitro treatment of IL34-KO SCs and WT SCs with supernatant medium

To investigate myofiber-transcribed IL34 could affect fates of SCs, isolated WT and IL34-KO SCs were first cultured in growth medium, and cells at 70% confluence were induced to differentiate by switching growth medium to differentiation medium. Collecting supernatant medium from 2-day differentiated WT and IL34 cultures, immediately adding them into IL34-KO and WT proliferating SCs and allowing them to grow for 24 h.

### In vitro manipulation of STAT3 activity

To disrupt STAT3 function in cultured SCs, isolated WT myoblasts from the adult mice were initially cultured in growth medium then induced to differentiation by switching growth medium into differentiation medium with 2 μM Stattic (Med Chem Express) for 48 h. Cells cultured in differentiation medium containing the same volume of DMSO were used as control groups.

To force STAT3 function in cultured SCs, isolated WT myoblasts from the adult mice were initially cultured in growth medium then induced to differentiation by switching growth medium into differentiation medium with 50 ng/ml recombinant Oncostatin M (OSM, Origene); the same volume of 0.1% BSA was used as a control. After OSM or BSA-treatment for 24 h, samples were collected for further analysis.

### Quantitative PCR

Total RNA from SCs or muscles was extracted with TRIzol reagent (Invitrogen, Life Technologies) according to the manufacturer's instructions. cDNA synthesis for PCR analyses was performed with 1 μg of total RNA at 25°C for 10 min, 42°C for 15 min and 85°C for 5 min by using 5× All-In-One RT MasterMix (ABM Biotech). Real-time PCR analysis was performed using 2× RealStar Green Power Mixture (Gene-Star). The qRT-PCR primers were as follows: IL34-forward: 5'-TACAAGAACCGGCTTCAGTACA-3'; IL34-reverse: 5'-GCATTGAGACTCACCAAGACC-3'; GAPDH-forward: 5'-CCCAGAAGACTGTGGATGG-3'; GAPDH-reverse: 5'-ACACATTGGGGGTAGGAACA-3'; Igfbp5-forward: 5'-CCCTGCGACGAGAAAGCTC-3'; and Igfbp5-reverse: 5'-GCTCTTTTCGTTGAGGCAAACC-3'.

### Transfection and luciferase assays

The promoter region containing NFKB1 binding sites of Igfbp5 and its variants with mutations in NFKB1 binding sites was cloned into the pGL3-basic vector (renamed Igfbp5-WT and Igfbp5-mut). The wild-type and mutant plasmids were electrotransfected with the phRL-TK control plasmid to C2C12 cells. A Dual-Glo luciferase assay kit (Promega) was used to measure the luciferase activity of firefly and Renilla 48 h after transfection according to the manufacturer's instructions.

### Statistical analysis

At least three replicates were applied in all experiments. Values are presented as the mean ± SEM. The statistical significance of differences between two means was calculated with a t-test. P<0.05 was considered to be significant.

## Supplementary Material

Supplementary figures.

## Abbreviations

IL34: interleukin 34
SCs: satellite cells
PAF: paraformaldehyde
CSA: cross-sectional area
Igfbp5: insulin-like growth factor binding protein-5
IP: intraperitoneal
NFKB1: nuclear factor of kappa light polypeptide gene enhancer in B-cells 1
AKT: Protein Kinase B
