# Supplementary material for: Inactivating IL34 promotes regenerating muscle stem cell expansion and attenuates Duchenne muscular dystrophy in mouse models
Source: Theranostics. 2023 Apr 23;13(8):2588–604. doi: 10.7150/thno.83817 (PMC10196826; doi:10.7150/thno.83817)
Supplement: Supplementary file 1 — Supplementary figures. [file thnov13p2588s1.pdf]

Supplementary Information

Inactivating IL34 promotes regenerating muscle stem cell expansion and attenuates Duchenne muscular dystrophy in mouse

Yang Su[1,2,3#], Yuxin Cao[1#], Chang Liu[1], Qing Xu[1], Na Li[1], Miaomiao Lan[1], Lei Li[1], Kun Wang[1] , Zeyu Zhang[3], and Qingyong Meng[1,4,✉]

1. State Key Laboratories for Agrobiotechnology, College of Biological Sciences, China Agricultural University, Yuanmingyuan West Road No. 2, Haidian District, Beijing 100193, China

2. Department of Cell Biology, Third Military Medical University (Army Medical University), Gaotanyan Road No. 30, Shapingba District, Chongqing 400038, China

3. State Key Lab of Animal Nutrition, College of Animal Science and Technology, China Agricultural University, Yuanmingyuan West Road No. 2, Haidian District, Beijing 100193, China

4. Beijing Advanced Innovation Center for Food Nutrition and Human Health, College of Biological Sciences, China Agricultural University, Yuanmingyuan West Road No. 2, Haidian District, Beijing 100193, China

✉ Corresponding authors: qymeng@cau.edu.cn

[#] Yang Su and Yuxin Cao contributed equally to this work.

Figure S1

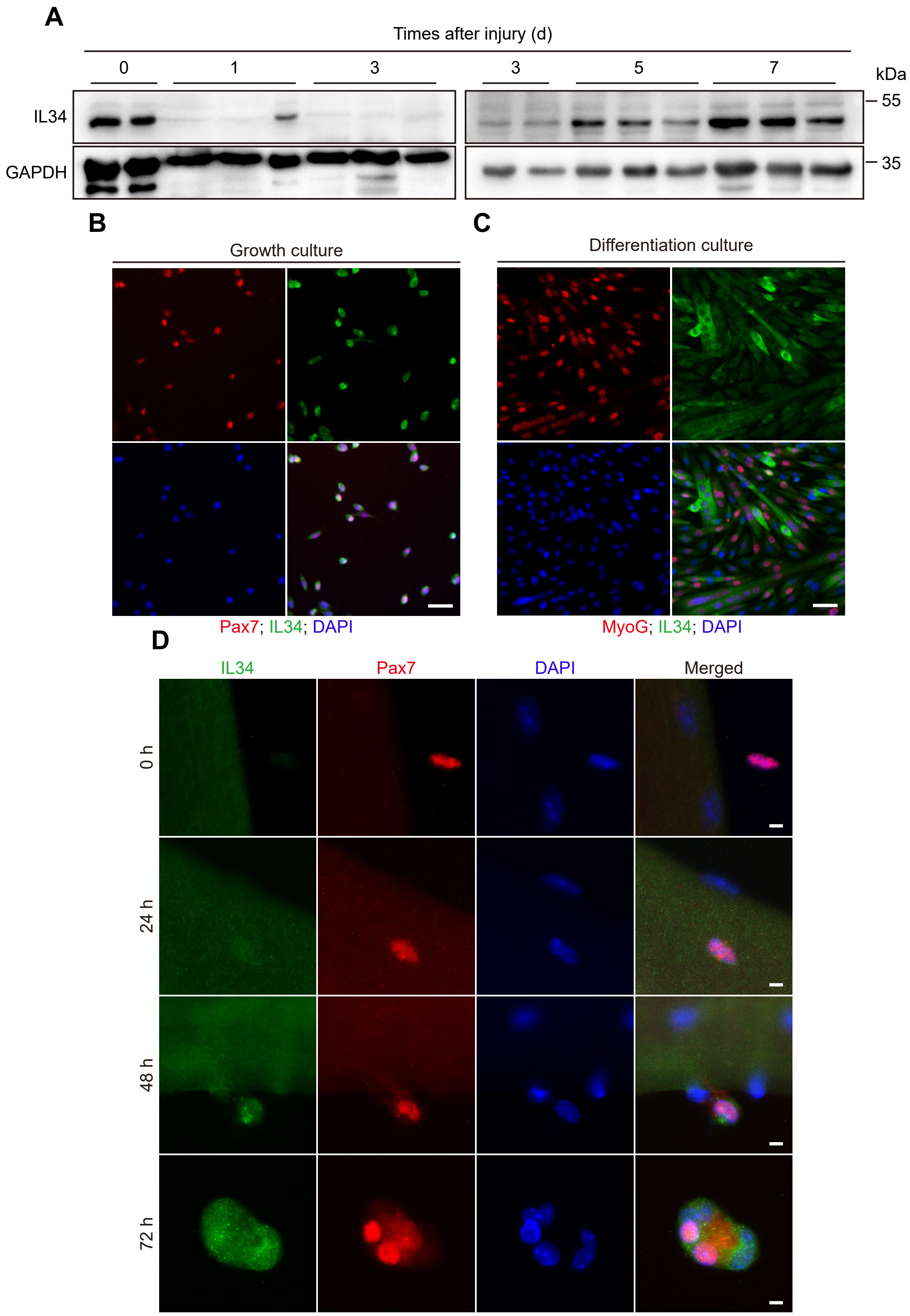

A
Times after injury (d)
0
1
3
3
5
7
kDa
IL34
GAPDH
55
35
B
Growth culture
Pax7; IL34; DAPI
C
Differentiation culture
MyoG; IL34; DAPI
D
IL34
Pax7
DAPI
Merged
0 h
24 h
48 h
72 h

Figure S1. Expression patterns of IL34 during in vitro myogenesis and muscle regeneration. **(A)** Western blot showing the protein levels of IL34 and unrelated β-tubulin at various stages of skeletal muscle regeneration. **(B)** Cells undergoing growth culture were fixed and labeled with anti-Pax7 and anti-IL34 antibodies. DAPI was used to identify nuclei. Representative individual and overlaid images of WT cultures after labeling with Pax7, IL34 and DAPI. Scale bars: 30 μm. **(C)** Cells induced to differentiate were fixed and labeled with anti-MyoG and anti-IL34 antibodies. DAPI was used to identify nuclei. Representative individual and overlaid images of WT cultures after staining for MyoG, IL34 and DAPI. Scale bars: 30 μm. **(D)** IL34 immunofluorescence in $Pax7^{+}$ SCs attached to freshly isolated EDL myofibers (0 h) or after culture for 24-72 h. Scale bar: 5 μm.

Figure S2

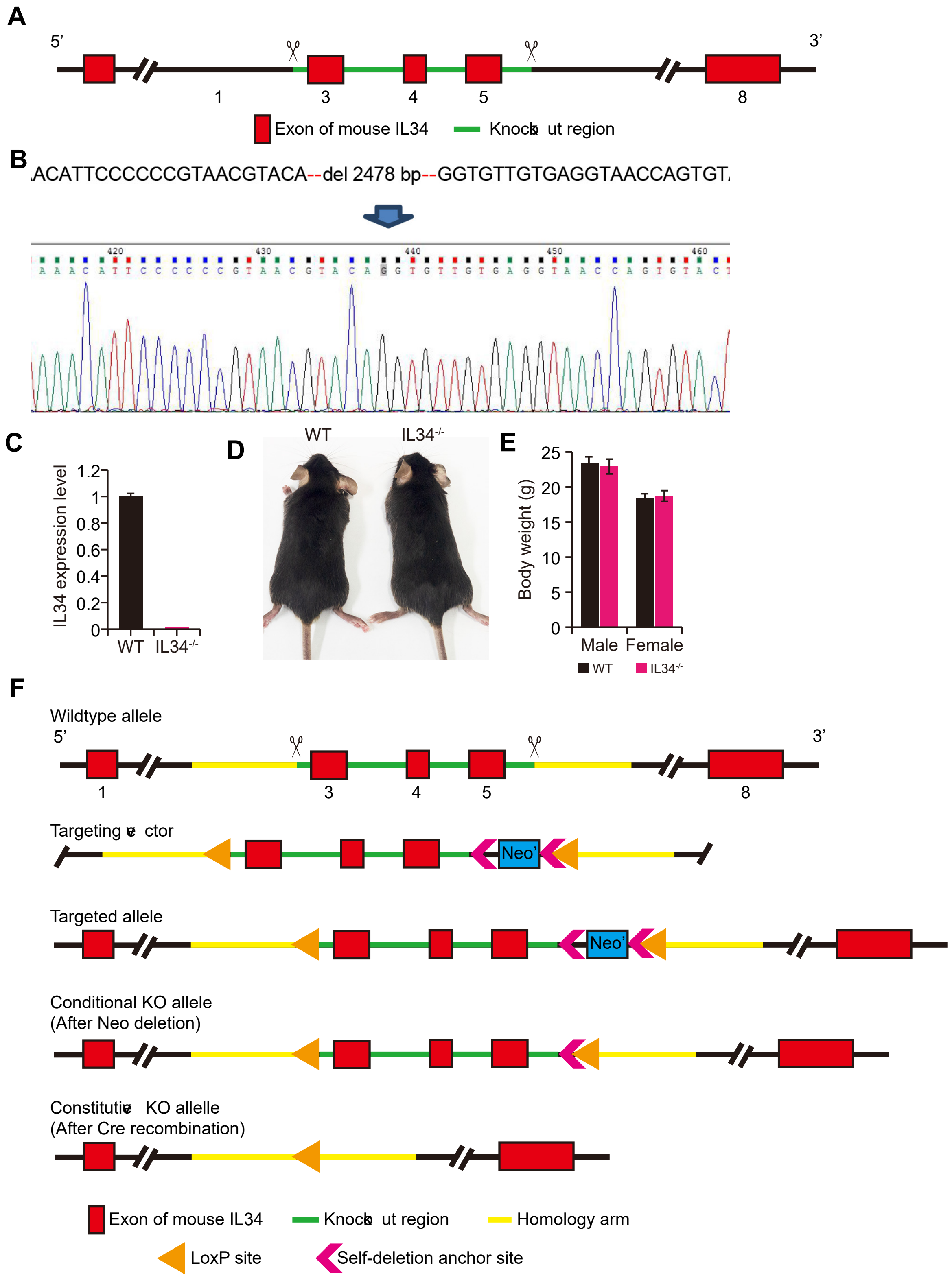
A
5'
3'
1
3
4
5
8
Exon of mouse IL34
Knockout region
B
ACATTCCCCCCGTAACGTACA--del 2478 bp--GGTGTTGTGAGGTAACCAGTGT
420
430
440
450
460
C
IL34 expression level
1.2
1
0.8
0.6
0.4
0.2
0
WT
IL34-/-
D
WT
IL34-/-
E
Body weight (g)
25
20
15
10
5
0
Male
Female
WT
IL34-/-
F
Wildtype allele
5'
3'
1
3
4
5
8
Targeting vector
Neo^r
Targeted allele
Neo^r
Conditional KO allele
(After Neo deletion)
Constitutive KO allelle
(After Cre recombination)
Exon of mouse IL34
Knockout region
Homology arm
LoxP site
Self-deletion anchor site

Figure S2. Generation of IL34 gene knockout mice. (A) Gene-targeting strategy for the generation of IL34 constitutive knockout mice. **(B)** DNA sequencing detecting the IL34 gene fragment that was effectively depleted in the mouse genome. **(C)** IL34 expression level in adult WT mice and IL34 gene deletion mice. **(D)** Comparison of adult WT mice and $IL34^{-/-}$ mice. **(E)** Measurement of body weight in WT and $IL34^{-/-}$ mice. N=5 mice in each group. **(F)** Gene-targeting strategy for the generation of IL34 gene conditional knockout mice.

Figure S3

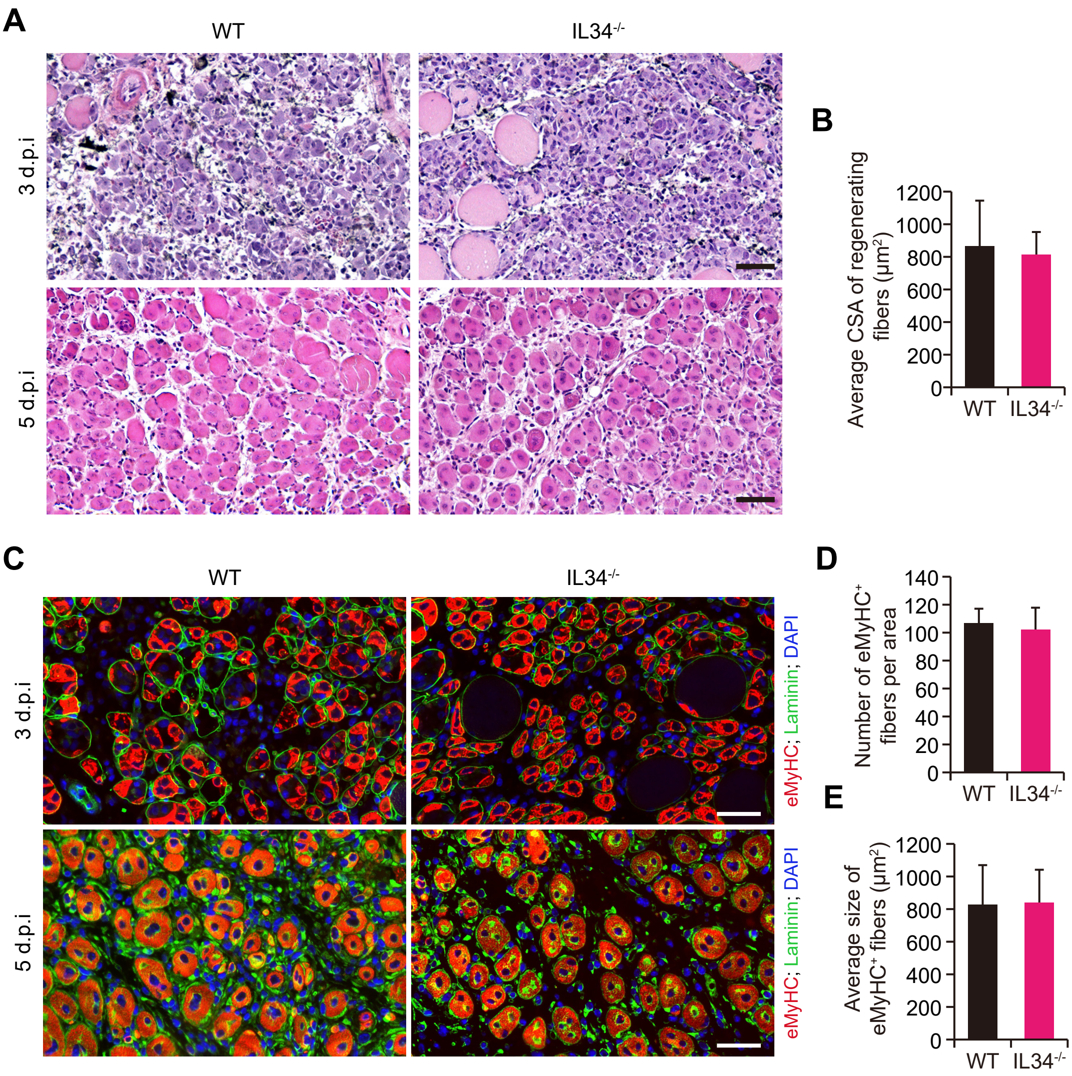

A
WT
IL34-/-
3 d.p.i
5 d.p.i
B
Average CSA of regenerating fibers (μm²)
1200
1000
800
600
400
200
0
WT
IL34-/-
C
WT
IL34-/-
3 d.p.i
5 d.p.i
eMyHC; Laminin; DAPI
eMyHC; Laminin; DAPI
D
Number of eMyHC+ fibers per area
140
120
100
80
60
40
20
0
WT
IL34-/-
E
Average size of eMyHC+ fibers (μm²)
1200
1000
800
600
400
200
0
WT
IL34-/-

Figure S3. IL34 has weak effects on the early stage of skeletal muscle regeneration. **(A)** H&E staining of transverse sections of TA muscle 3 and 5 days after injury. Scale bar, 50 μm. **(B)** Quantification of the average CSA of nuclei-centralized myofibers in 5-day postinjury TA muscle from WT and IL34 KO mice. **(C)** Representative merged photomicrographs of transverse sections of TA muscle 3 and 5 days after injury immunostained for eMyHC, laminin and DAPI. Scale bar, 50 μm. **(D)** Quantification of the number of $eMyHC^+$ cells per area in 3-day postinjury transverse sections of TA muscle. **(E)** Measurement of the average CSA of $eMyHC^+$ cells in transverse sections of the TA muscle 5 days postinjury.

Figure S4

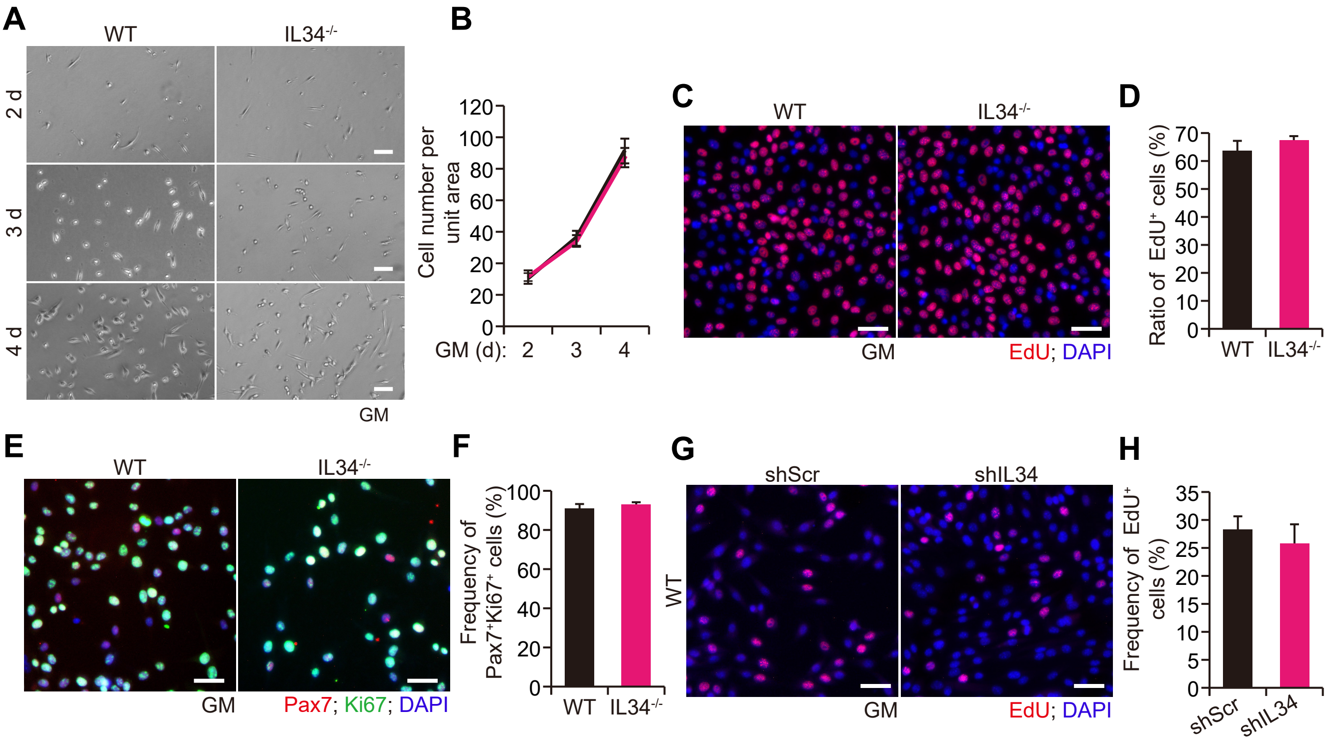
A
WT
IL34-/-
2 d
3 d
4 d
GM
B
Cell number per unit area
120
100
80
60
40
20
20
0
GM (d): 2 3 4
C
WT
IL34-/-
GM
EdU; DAPI
D
Ratio of EdU+ cells (%)
70
60
50
40
30
20
10
0
WT IL34-/-
E
WT
IL34-/-
GM
Pax7; Ki67; DAPI
F
Frequency of Pax7+Ki67+ cells (%)
100
80
60
40
20
0
WT IL34-/-
G
shScr
shIL34
WT
GM
EdU; DAPI
H
Frequency of EdU+ cells (%)
35
30
25
20
15
10
5
0
shScr
shIL34

Figure S4. Proliferation assays of WT and IL34-KO SCs. (A) Approximately 4×10^4 FACS-isolated WT and IL34-KO SCs were plated in 12-well plates precoated with Matrigel and cultured in growth medium. Representative photomicrographs of SCs grown for 2 days, 3 days, and 4 days. Scale bar, 100 μm. **(B)** Quantification of the growth rate of WT and IL34-KO SCs in growth medium. **(C)** SCs were incubated in growth medium. Representative overlaid images of expanded WT and IL34-KO cultures after labeling with EdU. Nuclei were costained with DAPI. Scale bar: 30 μm. **(D)** Quantification of the frequency of EdU^+ cells in WT and IL34-KO myoblasts. **(E)** Representative merged images of WT and IL34-KO myoblasts after immunostaining with Pax7, Ki67 and DAPI. Scale bar: 30 μm. **(F)** Quantification ratio of $Pax7^+Ki67^+$ cells in WT and IL34-KO SCs cultured in growth medium. **(G)** Representative merged images of WT myoblasts infected with lentivirus expressing shIL34 or shScr after labeling with EdU and DAPI. Scale bar: 30 μm. **(H)** Quantification ratio of EdU^+ cells in WT myoblasts interfering with IL34.

Figure S5

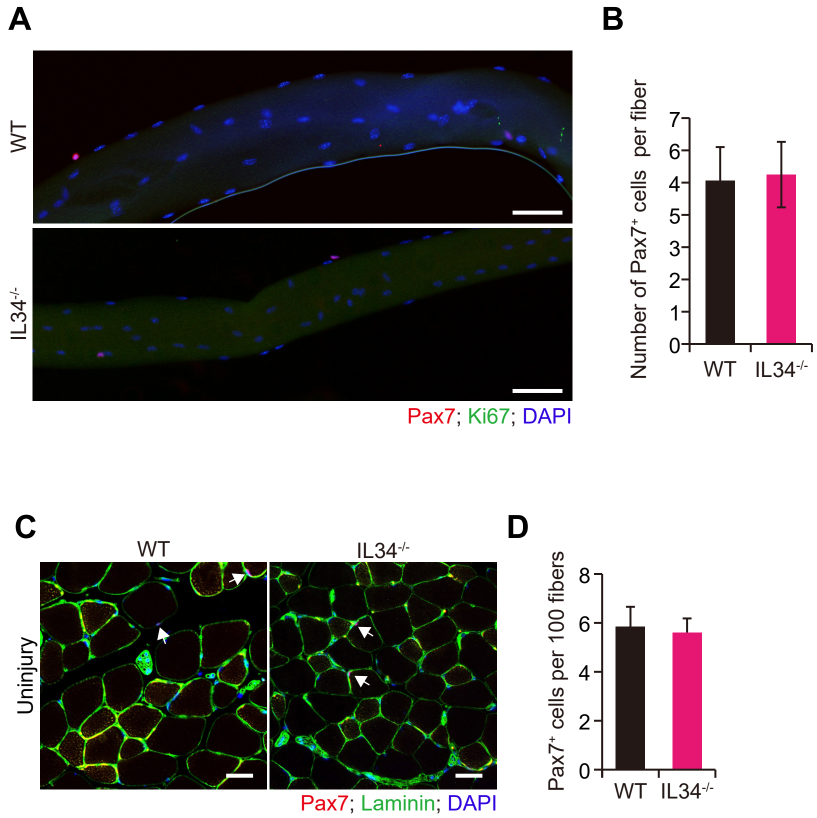
A
WT
IL34-/-
Pax7; Ki67; DAPI
B
Number of Pax7+ cells per fiber
7
6
4
5
3
2
1
0
WT
IL34-/-
C
WT
IL34-/-
Uninjury
Pax7; Laminin; DAPI
D
Pax7+ cells per 100 fibers
8
6
4
2
0
WT
IL34-/-

Figure S5. Inhibition of IL34 has no effects on the number of Pax7$^+$ cells in freshly isolated single myofibers and transverse sections of uninjured skeletal muscle. (A) Representative merged photomicrographs of freshly isolated WT and IL34-KO single myofibers after labeling for Pax7, Ki67 and DAPI. Scale bar: 50 μm. **(B)** Quantitative estimation of the number of Pax7$^+$ cells on freshly isolated single myofibers. **(C)** Representative merged photomicrographs of normal TA muscle transverse sections labeled for Pax7, laminin and DAPI. Scale bar: 30 μm. **(D)** Percentage of Pax7$^+$ cells (normalized to myofibers) in transverse sections of uninjured TA muscle from WT and IL34-KO mice.

Figure S6

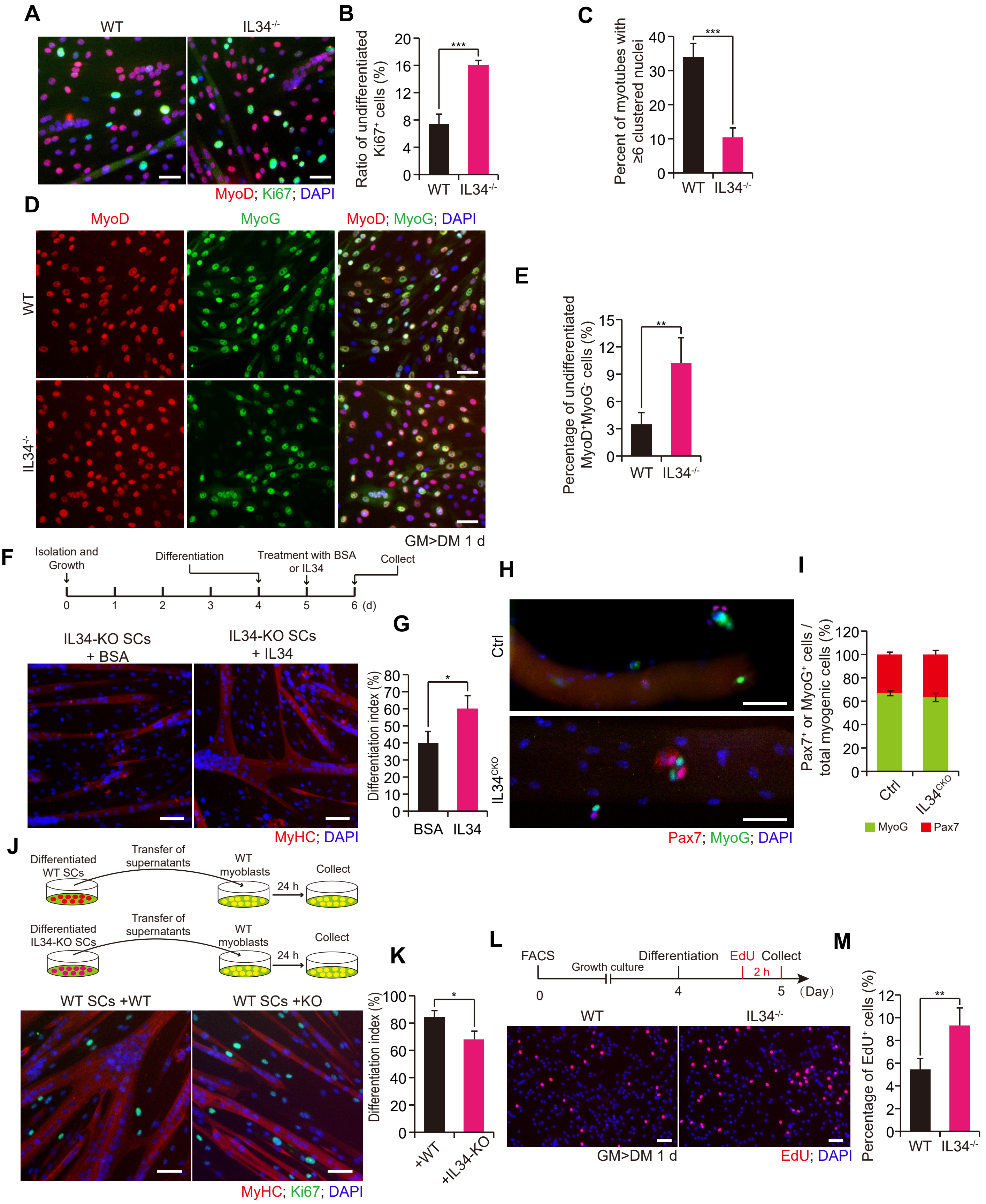
A
WT
IL34-/-
MyoD; Ki67; DAPI
B
Ratio of undifferentiated Ki67+ cells (%)
***
WT IL34-/-
C
Percent of myotubes with ≥6 clustered nuclei
***
WT IL34-/-
D
MyoD
MyoG
MyoD; MyoG; DAPI
WT
IL34-/-
GM>DM 1 d
E
Percentage of undifferentiated MyoD+MyoG- cells (%)
**
WT IL34-/-
F
Isolation and Growth
Differentiation
Treatment with BSA or IL34
Collect
0 1 2 3 4 5 6 (d)
IL34-KO SCs + BSA
IL34-KO SCs + IL34
MyHC; DAPI
G
Differentiation index (%)
*
BSA IL34
H
Ctrl
IL34CKO
Pax7; MyoG; DAPI
I
Pax7+ or MyoG+ cells / total myogenic cells (%)
Ctrl
IL34CKO
MyoG
Pax7
J
Differentiated WT SCs
Transfer of supernatants
WT myoblasts
24 h
Collect
Differentiated IL34-KO SCs
WT SCs +WT
WT SCs +KO
MyHC; Ki67; DAPI
K
Differentiation index (%)
*
+WT
+IL34-KO
L
FACS
Growth culture
Differentiation
EdU
Collect
2 h
0 4 5 (Day)
WT
IL34-/-
GM>DM 1 d
EdU; DAPI
M
Percentage of EdU+ cells (%)
**
WT IL34-/-

Figure S6. IL34 promotes SC differentiation. **(A)** WT and IL34-KO SCs were cultured in 10% horse serum medium for 3 day. The cells were then fixed and labeled with MyoD, Ki67 and DAPI. Representative merged photomicrographs of WT and IL34-KO cultures after labeling with MyoD, Ki67 and DAPI. Scale bars: 30 μm. **(B)** Quantitation of the percentage of undifferentiated $Ki67^+$ cells in WT and IL34-KO cultures. ***P<0.001. **(C)** Percentage of $MyHC^+$ fibers containing ≥ 6 nuclei relative to total $MyHC^+$ fibers. ***p<0.001 (Related to Figure 2E and F). **(D)** SCs were initially incubated in growth medium, and then the growth medium was switched to differentiation medium. The cells were then fixed and labeled with anti-MyoG, anti-MyoD and DAPI. Representative overlaid images of WT and IL34-KO cultures after labeling with MyoG, MyoD and DAPI. Scale bars: 30 μm. **(E)** Quantitative estimation of the percentage of undifferentiated $MyoD^+MyoG^-$ cells in WT and IL34-KO cultures. **P<0.01. **(F)** One-day differentiated IL34-KO SCs were treated with BSA or IL34 recombinants, and representative merged images of cultures were costained with MyHC and DAPI. Scale bar: 50 μm. **(G)** Measurement of the differentiation index was calculated by the frequency of nuclei in $MyHC^+$ cells relative to total nuclei. *p<0.05. **(H)** Representative overlapping images of 72 h cultured myofibers from the EDL muscle of Ctrl and $IL34^{CKO}$ mice costained for Pax7, MyoG and DAPI. Scale bar: 50 μm. **(I)** Percentage of $Pax7^+MyoG^-$ and $Pax7^-MyoG^+$ cell populations in Ctrl and $IL34^{CKO}$ myofiber-associated SCs. **(J)** WT myoblasts induced to differentiation by supplementing with supernatant medium collected from differentiated WT or IL34-KO SCs, respectively. Representative merged images of cultures were costained with

MyHC, Ki67 and DAPI. Scale bar: 50 μm. **(K)** Measurement of the differentiation index was calculated by the frequency of nuclei in $MyHC^+$ cells relative to total nuclei. *p<0.05. **(L)** After incubation in growth medium, cells were induced to differentiate for 1 day, and EdU was added to label proliferating cells for the last 2 h. The cells were then fixed and analyzed by staining with EdU and DAPI. Representative merged photomicrographs of WT and IL34-KO cultures after staining with EdU and DAPI. Scale bars: 50 μm. **(M)** Proportion of EdU^+ cells in WT and IL34-KO cultures. **p<0.01.

Figure S7

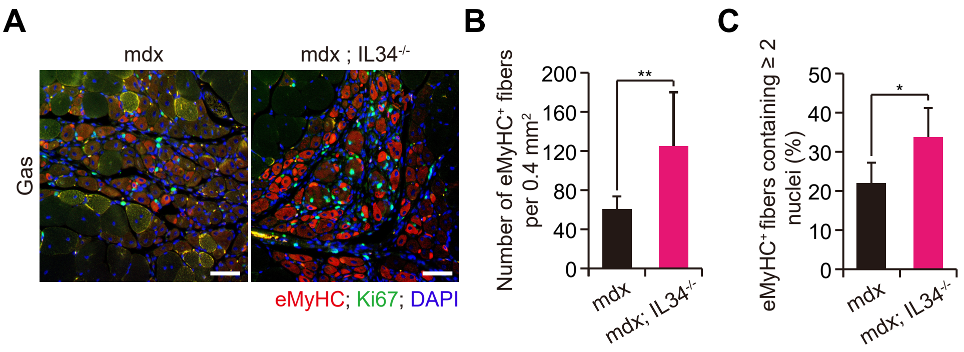
A
mdx
mdx ; IL34-/-
Gas
eMyHC; Ki67; DAPI
B
Number of eMyHC+ fibers per 0.4 mm2
200
160
120
80
40
0
**
mdx
mdx; IL34-/-
C
eMyHC+ fibers containing ≥ 2 nuclei (%)
50
40
30
20
10
0
*
mdx
mdx; IL34-/-

Figure S7. Genetic inactivation of IL34 in mdx mice attenuates Duchenne muscular dystrophy. **(A)** Representative merged images of eMyHC and Ki67 coimmunostained Gas sections from mdx and mdx::IL34$^{-/-}$ mice at 12 weeks of age. Nuclei was labeled with DAPI. Scale bar: 30 μm. **(B)** Number of eMyHC^{+} cells per field. **p<0.01. **(C)** Percentage of eMyHC^{+} fibers containing ≥ 2 nuclei relative to total eMyHC^{+} fibers. *p<0.05.
